# Wide-field optical imaging of electrical charge and chemical reactions at the solid–liquid interface

**DOI:** 10.1073/pnas.2209955119

**Published:** 2022-12-02

**Authors:** Sushanta Mahanta, Pedro Vallejo-Ramirez, Narain Karedla, Paweł Puczkarski, Madhavi Krishnan

**Affiliations:** ^a^Physical and Theoretical Chemistry Laboratory, Department of Chemistry, University of Oxford, Oxford OX1 3QZ, United Kingdom; ^b^The Kavli Institute for Nanoscience Discovery, Oxford OX1 3QU, United Kingdom

**Keywords:** electrical potential and surface charge measurement, interface characterization, surface chemistry, thin-film properties, electrostatic imaging

## Abstract

From molecules and particles to macroscopic surfaces immersed in fluids, chemical reactions often endow interfaces with electrical charge which in turn governs surface interactions and interfacial phenomena. The ability to measure the electrical properties of a material immersed in any solvent, as well as to monitor the spatial heterogeneity and temporal variation thereof, has been a long-standing challenge. Here, we describe an optical microscopy-based approach to probe the surface charge distribution of a range of materials, including inorganic oxide, polymer, and polyelectrolyte films, in contact with a fluid. The method relies on optical visualization of the electrical repulsion between diffusing charged probe molecules and the unknown surface to be characterized. Rapid image-based measurements enable us to further determine isoelectric points of the material as well as properties of its ionizable chemical groups. We further demonstrate the ability to optically monitor chemically triggered surface charge changes with millisecond time resolution. Finally, we present a scanning-surface probe technique capable of diffraction-limited imaging of spatial heterogeneities in chemical composition and charge over large areas. This technique will enable facile characterization of the solid–liquid interface with wide-ranging relevance across application areas from biology to engineering.

## Significance

The solid–liquid interface is an important entity across disciplines. The ability to characterize interfacial properties can prove decisive in a range of vital application areas including pharmaceutical formulations, catalysts and energy production, and storage devices. The electrical charge that develops at the solid–liquid interface exerts a profound influence on surface dynamics and interactions, and yet we have few, if any, broadly applicable techniques that permit interfaces and their properties to be interrogated in a straightforward fashion. Here we demonstrate the ability to perform simple, wide-field optical imaging of surface electrical charge at the spatial resolution of the optical microscope. This fosters large-area (millimeter scale), direct visualization of surface chemistry and time-dependent changes due to chemical reactions at surfaces.

The electrical properties of a surface in contact with an electrolyte, and detailed knowledge thereof, play a fundamental role across fields in science and engineering, and in application areas as diverse as water purification, anti-fouling and antimicrobial coatings, corrosion prevention, pharmaceuticals, diagnostic devices, fine chemicals and drug delivery, and energy storage, to name a few (1–4). Many experimental techniques to measure electrical charge at the solid–gas interface provide excellent measurement sensitivity at a high spatial resolution, but they often require ultrahigh vacuum conditions and low temperatures (<2 K) and cannot be applied to solid interfaces in contact with fluid at room temperature (5–9).

In solution, the ionization of chemical groups carried by an object or a surface—via ion dissociation or association reactions—results in a net electrical charge density that is characteristic of the material’s chemistry, but can be strongly governed by the pH and salt concentration in solution. Whereas surface characterization techniques that rely on electrokinetic phenomena and electronic detection can provide area-averaged readouts of surface charge values (10, 11), scanning probe-based techniques such as chemical and electrochemical force microscopy, and scanning ion conductance microscopy have sought to address the measurement of spatial heterogeneity of charge and chemical composition at interfaces in solution (12, 13). Kelvin Probe Force Microscopy can be applied to surface measurements in weak electrolytes, but it is generally only applicable to interfaces in contact with apolar solvents (12, 14). Furthermore, methods that require mechanical scanning of a probe object over the surface of interest are generally limited to relatively low imaging rates of ≈1 μm^2^/s. Alternatively, modifications to traditional X-ray photoelectron spectroscopy methods support the measurement of electrical surface potentials at the solid–liquid interface as well as potential distributions in the electrical double layer (15, 16), but the requirement of an X-ray source hinders the broad availability of such techniques. Recently, optical imaging techniques have emerged as a powerful route to addressing the measurement of surface ionization at the solid–liquid interface. Here, molecules adsorbed to surface groups, or water molecules in the immediate vicinity of a charged surface, act as probes of the local electrical field and generate optical signals that provide information on the solid–liquid interface (16–20). Moving away from the immediate vicinity of the interface further into bulk solution, studies have shown that the local concentration of nanoscale entities—such as colloidal particles, vesicles, and fluorescent dyes—diffusing in solution, can itself be harnessed to shed light on the properties of the probe and its interaction with the solid–liquid interface (21–27). Nanoscale entities dispersed in solution thus act as “thermally scanned” probes of an interaction with a surface and may thereby serve to report on properties of the solid–liquid interface.

In previous work, we used parallel plate systems composed of silica and glass surfaces separated by a defined gap height in order to precisely measure the effective charge of a range of molecular species and colloidal particles both in organic solvents and in aqueous solution (27–32). Using a molecular species of known effective charge, we then determined the electrical charge density and potential of the silica surfaces in the nanoslit devices (31, 33, 34). In this study, we describe a general method to measure the surface electrical properties of an ionizable material immersed in a solvent. The approach relies on optical snapshots of an ensemble of light-emitting molecules of known effective electrical charge diffusing in solution. The technique uses a standard wide-field fluorescence microscope and can support imaging over millimeter lengths of substrate with diffraction-limited spatial resolution (based on a spatially scannable single field of view of approximately 0.25 mm × 0.25 mm). This corresponds to an imaging rate of approximately 10^6^ μm^2^/s which is roughly a million times faster than cantilever based techniques.

**Fig. 1.**
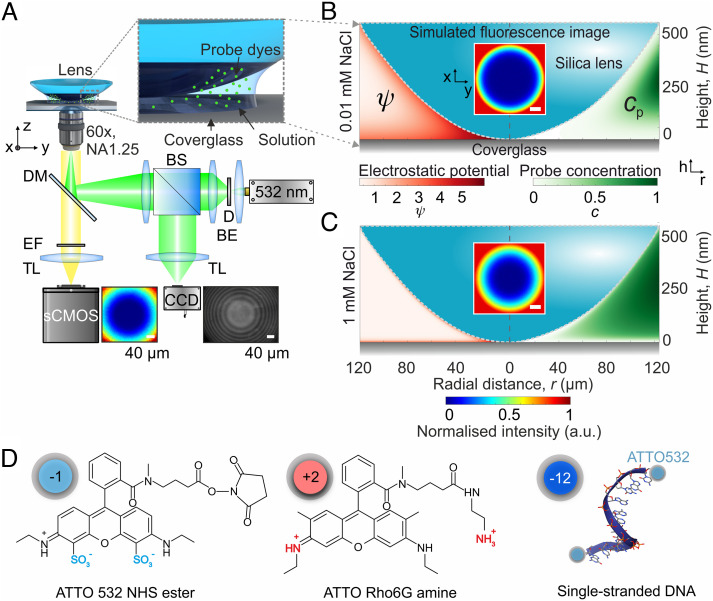
Principle underlying optical imaging of surface electrical charge. (*A*) Layout of the wide-field fluorescence microscope-based measurement setup. D, diffuser; BE, beam expander; BS, beam splitter; TL, tube lens; DM, dichroic mirror; EF, emission filter. The *Inset* presents a schematic of the variable-height gap created between the surfaces of a borosilicate lens and glass substrate, with charged fluorescent probe molecules (green spheres) in solution. (*B*,*C*) Schematic depiction of the variable height gap geometry up to a maximum height of 500 nm, overlaid with calculated heatmaps of the electrostatic surface potential (*Left*), calculated using the PB equation for surface charge density $\sigma =0.025\;e/{\mathrm{nm}}^{2}$ (*SI Appendix*), and the corresponding normalized probe concentration (*Right*) in electrolytes containing NaCl at concentrations of c=0.01 mM (*B*) and 1 mM (*C*). *Inset* images present calculated fluorescence intensity distributions expected in our imaging system. (*D*) Chemical structure and structural charge, $q_{str}$, of the fluorescent probe molecules used in this work. The scale bar in all fluorescent images corresponds to 40 μm.

In order to illustrate an application of our measurement principle to the characterization of the surface properties of borosilicate glass in contact with aqueous electrolyte, we use a lens-based confinement system previously utilized in a variety of optical studies on molecules and particles in solution (25, 35–38). The system consists of a fluid-filled gap of variable height created between a convex lens and a flat glass substrate (Fig. 1*A*), at least one of which can be coated with an unknown material whose surface properties are to be determined. We introduce charged, light-emitting probe molecules in solution into the gap (Fig. 1*D*), and image the spatial optical intensity distribution resulting from the thermal motion of probe molecules sampling the gap. Morrin et al. have previously shown that the spatial distribution of fluorescence intensity in such a convex lens–coverglass system can serve to measure the local height of the gap (25). Furthermore, the study also demonstrated that electrostatic effects could make substantial contributions to measured local optical intensities.

Contact with the fluid causes the ionizable groups on the two flanking surfaces to acquire a net electrical charge density, *σ*, which is associated with an electrical surface potential, $\phi_{s}$, shown schematically in Fig. 1 *B* and *C* and *SI Appendix*, Fig. S2. In the limit of low magnitudes of *σ*, the electrical potential at any radial location, r, in the gap can be written in a simplified form as:


[1] $$\phi (r,z)\approx \phi_{s,1}e^{-\kappa z}+\phi_{s,2}e^{-\kappa (H-z)},$$

where $\phi_{s,1}$ and $\phi_{s,2}$ denote effective electrical surface potentials at each of the two surfaces (Fig. 1 *B* and *C*), z is the local axial coordinate in the gap (Fig. 1*A*), and $H\left(r\right)$ is the radially dependent height of the gap which is measured directly using optical interferometry (*SI Appendix*, Fig. S1). Furthermore $\kappa^{-1}=\sqrt{\frac{\varepsilon_{0}\varepsilon_{m}k_{B}T}{2N_{A}ce^{2}}}$ is the Debye length, where c is the concentration of monovalent salt in solution, *e* is the elementary charge, $N_{A}$ is Avogadro’s number, $\varepsilon_{m}=78.5$ is the dielectric constant of water, and $\varepsilon_{0}$ is the permittivity of free space. The concentration, $c_{p}$, of the charged probe molecule anywhere in the gap is then given by the Boltzmann distribution (28, 29),


[2] $$c_{p}\left(r,z\right)=c_{p,0}e^{-\frac{U(r,\;z)}{k_{B}T}},$$

where $c_{p,0}$ is the concentration of the probe molecule in bulk solution (Fig. 1 *B* and *C*) and $U\left(r,,,z\right)=q_{eff}\phi \left(r,,,z\right)$ is the local axial electrostatic energy of a probe molecule of effective charge $q_{eff}$ (27, 28, 39, 40). Importantly, we have previously shown that the effective charge of an object in solution, $q_{eff}$, is not necessarily equal to its bare or structural charge, $q_{str}$, which in turn is given by the sum of charge carried by its ionizable groups and any bound ions (27, 28, 39–42). For weakly charged molecules whose structural charge $|q_{str}|≲3e$, we have $q_{eff}≅q_{str}$. But, in general, $q_{eff}<q_{str}$, which is important to take into account in order to achieve quantitatively accurate measurements of $\phi_{s}$ (27, 28).

The measured local optical intensity at a radial location, *r*, in an image is then:


[3] $$I\left(r\right)=\beta c_{p,0}\int_{0}^{H}e^{\frac{-U(r,\;z)}{k_{B}T}}dz.$$

In this work, we use probe molecules in solution at concentrations ranging from $c_{p,0}=100$ nM to 100 µM, which yield high SNR snapshots at a time resolution ranging from 10 s down to ≈1 ms. Because we work with an arbitrary proportionality constant β (*SI Appendix*) in Eq. **3**, our results are independent of the bulk probe molecule concentration, $c_{p,0}$, which is a constant in any given experiment (*SI Appendix*).

**Fig. 2.**
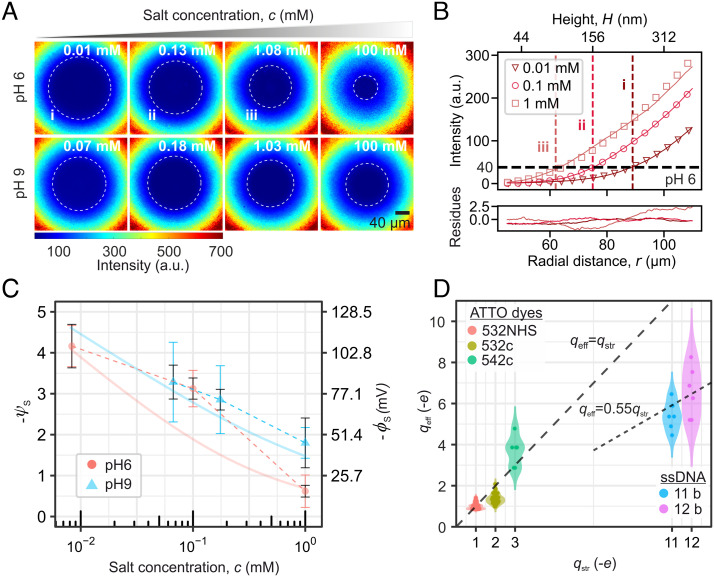
Characterizing the surface electrical and chemical properties of silica. (*A*) Experimentally recorded images of the fluorescent intensity distribution of ATTO 532NHS dye $(q_{str}=-1e$) in solutions of varying ionic strength, from c=0.01 mM to 100 mM NaCl, measured at pH ≈ 6 and pH ≈ 9. The white dashed circle represents a contour line at 40 counts which provides a comparative measure of the strength of the electrostatic repulsion across various conditions. The 40-μm scale bar is the same for all images. (*B*) Plot of the azimuthally averaged radial intensity profiles (symbols) for three values of ionic strength at pH ≈ 6 shown in (*A*), with corresponding theoretically calculated intensity curves (solid lines) accompanied by a plot of residues below. Theoretically modeled radial intensity curves yield values of $\psi_{s}$ for each experiment. Vertical dashed lines denote a radius of fixed intensity across the images and correspond to the circles in the images in (*A*). At higher salt concentrations, or lower surface charge densities, the dye penetrates regions of smaller radii, reducing the extent of the visually evident exclusion zone. (*C*) Surface electrical potentials (closed symbols) estimated from images recorded at c=0.01, 0.1, and 1 mM NaCl shown in (*A*). Colored error bars represent the SD under each measurement condition, including sample-to-sample variation (*SI Appendix*,* *Fig. S6). Black error bars display the uncertainty due to the measurement approach alone (*SI Appendix*,* *Fig. S6). Solid lines represent calculation of $\psi_{s}$ using a chemical ionization model for silica for parameter values as shown in the table in *SI Appendix*,* *Fig. S8*E* and described in detail in the text. Dashed lines are visual guides. (*D*) Plot of the effective charge $q_{eff}$ vs. structural charge $q_{str}$ for five probe molecules: three weakly charged organic dyes ($\left|q_{str}\right|\leq 3e$), and two highly charged single-stranded DNA molecules ($\left|q_{str}\right|>5e$), measured using a known electrical surface potential of silica as presented in (*C*), with $\psi_{\text{s}}\approx$ -3 at 0.1 mM NaCl and $\psi_{\text{s}}\approx$ -2 at 1 mM NaCl and pH 9. Spatial intensity distributions and I(r) curves for the molecules are shown in *SI Appendix*,* *Fig. S9. DNA oligonucleotides display $q_{eff}$ < $q_{str}$ due to charge renormalization (28, 41). Note that we work with intensity profiles in the range r≥40 μm which excludes the contact region between the lens and the substrate.

Consider a gap created by electrically neutral surfaces so that the local electrical potential, $\phi \left(z\right)$, is zero everywhere. The probe molecule penetrates the gap uniformly and is at concentration $c_{p,0}$ throughout the system. The resulting image of optical intensity essentially captures the geometry of the variable-height gap (25, 38). In contrast, when both surfaces are homogeneously charged, the magnitude of the electrical potential in the gap is large and qualitatively in indirect relation to the local gap height. Like-charged probe molecules are therefore strongly repelled from regions where the confining surfaces are closer, and the local optical intensity is correspondingly low (25). Thus, we find that a single optical snapshot of the system yields an image which is radially symmetric, displaying a dark region at the center that we term the “exclusion zone” (*Insets* in Fig. 1 *B* and *C*). We compare measured optical intensities with calculations based on $\phi \left(r,,,z\right)$ obtained using the nonlinear Poisson–Boltzmann (PB) equation and Eqs. **2** and **3**, as described in *SI Appendix*. This comparison yields values for the surface electrical potential, $\phi_{s}$, or equivalently the surface charge density σ, revealing the electrical properties of the surface material (*SI Appendix*, Figs. S2 and S5). Note that in practice we do not rely on the approximation in Eq. **1** that is accurate only for very weakly charged surfaces where $e\phi_{s}\ll k_{B}T$. We further point out that the charge of the probe molecule is a useful tunable parameter in the measurement. We typically use weakly charged probe molecules such as singly or doubly charged organic fluorescent dyes ($q_{eff}=q_{str}=-1$*e* or −2e, Fig. 1*D*) in conjunction with strongly charged surfaces (e.g., $\left|\phi_{s}\right|\geq 2\;\frac{k_{B}T}{e}$ ≈ 50 mV). Weakly charged surfaces or surfaces measured in electrolytes where the salt concentration *c* is large are generally associated with a low magnitude of surface potential as $\left|\phi_{s}\right|\propto \left|\sigma_{s}\right|\propto 1/\sqrt{c}$. Here we find that strongly charged probes, e.g., single-stranded DNA molecules ($\left|q_{str}\right|>\left|q_{eff}\right|\geq 3e$) serve to enhance gradients in the intensity profiles (Fig. 2*D* and *SI Appendix*, Fig. S9).

In our analysis, if the charge of the probe molecule, $q_{eff}$, is known exactly, the only unknown is the surface electrical potential $\phi_{s}$ or surface charge density σ. Note that $\phi_{s}$ is generally larger in magnitude than, but is related to, the electrokinetic (ζ) potential measured in particle suspensions and in electro-osmotic flow-based surface measurements, as depicted in a diagram for the electrical double layer in *SI Appendix*, Fig. S2*A*. In reporting our measurements, we quote values of a dimensionless electrical potential $\psi_{s}={e\phi }_{s}/k_{B}T$ (43), with a value of $\psi_{s}=1$ corresponding to an electrical potential of $\phi_{s}\approx 25\;\mathrm{mV}$ at 298 K.

## Results

### Optical Image-Based Characterization of the Electrical and Chemical Properties of Silica Surfaces.

At the outset, we focus on electrical and chemical characterization of borosilicate glass surfaces. There is enormous interest in understanding the electrostatic properties of silica surfaces, as it is a critical material in chromatographic separations, adsorption, and transport of pollutants, as well as in bio-diagnostics and micro- and nanofluidic systems (17, 44, 45). The charge on silica surfaces arises from the dissociation of at least three different types of silanol groups, each associated with a different p*K*_a_ value, ranging from 3 to 10 (17, 18, 20, 46). Although the trimodal p*K*_a_ model seems to provide a sufficient description of the charging behavior of silica, nonlinear spectroscopy measurements have reported a continuous Gaussian distribution of p*K*_a_ values ranging from 2 to 11, with a mean value around 6.7 (20). In addition, the total number density of ionizable silanol groups is also highly variable, with reported values ranging from ≈1 to 8 nm^−2^. Moreover, the exact values of p*K*_a_, total number density and fractional amount of each type of group are further subject to variability based on sample synthesis and treatment history (46).

We performed a series of electrostatic imaging measurements under controlled pH and salt concentration conditions in a symmetric measurement configuration created by a borosilicate glass lens of radius of curvature 13.1 mm resting on top of a microscopy coverglass surface, where we assume $\phi_{s,1}=\phi_{s,2}=\phi_{s}$. We used negatively charged probe molecules (ATTO 532 NHS, $q_{str}=-1e$) in an electrolyte composed of deionized (DI) water containing variable amounts of added NaCl at final concentrations of c≈0.01, 0.1 mM, 1 mM, and 100 mM. Images of the dye distribution at pH 6 and 9 with varying salt concentration are shown in Fig. 2*A*. The decreasing radius of the exclusion zone with increasing salt concentration at both values of pH indicates an overall decrease in the magnitude of electrostatic repulsion between the dye and the flanking surfaces as expected. Leveraging the axial symmetry of the gap, we radially average the intensity distributions to obtain high signal-to-noise ratio (4 < SNR < 20) intensity profiles, I(r), which are then matched to calculations based on the nonlinear PB equation (Fig. 2*B* and *SI Appendix*, Figs. S1, S2 and S5), as described in *SI Appendix*. The results for the estimated surface potentials for silica are shown in Fig. 2*C*. For example, at pH ≈ 9, we measured effective surface potentials for silica in solutions containing c≈1 mM NaCl given by $\psi_{s}=-1.8\pm 0.6$ ($\sigma =-0.03\pm 0.01\;e/{\mathrm{nm}}^{2}$ ). However, at pH ≈ 6, the magnitude of the surface potential decreased significantly to a value $\psi_{s}=-0.6\;\pm 0.1$ ($\sigma =-0.007\pm 0.004\;e/{\mathrm{nm}}^{2}$) in 1 mM NaCl.

**Fig. 3.**
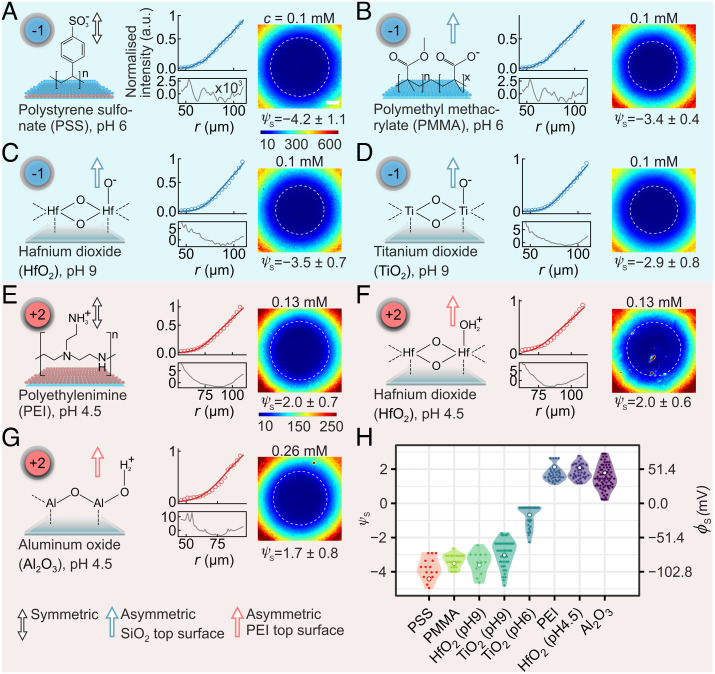
Measuring values of surface electrical potential for thin-film polymer, polyelectrolyte, and inorganic oxide coatings. (*A–G*) Measurements of surface electrical potential $\psi_{s}$ for positively and negatively charged materials. Individual panels display a representative image of the recorded fluorescence intensity distribution (*Right*), overlaid with a circular contour line at 40 counts to facilitate comparison across materials. The intensity scale shown in (*A*) is the same for all images in (*A*–*D*), and the scale shown in (*G*) is the same for (*E*–*G*). Also displayed are the chemical structures of the material of interest, coated on a coverglass substrate, as well as the structural charge, $q_{str}$, of the fluorescent probe molecule used in each case. Arrows denote either a symmetric measurement arrangement with the same material on both flanking surfaces (double arrowhead) or an asymmetric measurement system where the coating on the top surface was either silica or PEI ($\psi_{s,{SiO}_{2}}\approx -3.\;1$ for measurements at pH ≈ 6 and $\psi_{s,{SiO}_{2}}\approx -3.3$ for measurements at pH ≈ 9 at 0.1 mM NaCl; $\psi_{s,PEI}\approx 2$ at pH ≈ 4.5). Each plot displays a normalized measured I(r) curve and the corresponding theoretically modeled profile with a plot of residues below (shown rescaled by a factor 10^3^, factor only shown in (*A*). (*H*) Summary of measurements of $\psi_{s}$ values for all materials. Multiple data points for each case reflect variation arising from data analysis as well from sample-to-sample variation, as discussed in *SI Appendix, *Fig. S6. The mean value of the measured electrical surface potential of each material is presented as a white circle. Note that we only use intensity profiles in the range r≥40 μm which excludes the contact region between the lens and the substrate. The scale bar in (*A*) corresponds to 40 μm and all images have the same scale.

These measured values of $\psi_{s}$ can be further compared with a model for surface ionization in order to shed light on the chemistry of silica in the measurement (*SI Appendix*). The ionization probability, $\alpha_{i}$, of the *i*th species of ionizable groups, characterized by a dissociation constant corresponding to $pK_{i}$, is given by:


[4] $$\alpha_{i}=\frac{1}{1+10^{z_{i}\left(pH-pK_{i}\right)}e^{z_{i}\psi_{s}}},$$

where $z_{i}=\pm 1$ denotes the sign of the charge of the surface group in the ionized state (47). The total charge density, σ, on a surface composed of a mixture of groups of fractional density $f_{i}$, is then given by $\sigma =\sum_{i}z_{i}f_{i}\alpha_{i}\Gamma e$, where $\sum_{i}f_{i}=1$. The above equations imply that a higher pH results in greater ionization of acidic groups, leading to an increased charge density, σ, and thereby a larger magnitude of measured electrical potential, $\psi_{s}$, at the surface. Our measured values of $\psi_{s}$ and σ reflect these qualitative trends as shown in Fig. 2*C*. In order to glean the underlying chemical species model from the measured values of electrical potential, we compared our measurements of $\psi_{s}$ to values calculated for silica using a trimodal ionization model (Fig. 2*E* and *SI Appendix*, Fig. S8). We find that silica in our experiments is well described by a total silanol group number density of $\Gamma \approx 0.1\;{\mathrm{nm}}^{-2}$ comprising silanol species whose surface dissociation constants are given by p*K*_1_≤ 3_,_ p*K*_2_ = 7.2 ± 0.8 and p*K*_3_ = 9.7 ± 0.1 occurring at fractional densities $f_{1}=0.06\;\pm \;0.01$, $f_{2}=0.04\;\pm 0.01$ , and $f_{3}\approx 0.9$ (*SI Appendix*, Fig. S8). These values for the total group density and fractional species density agree with recent optical spectroscopic measurements as well as with our own previous single molecule measurements using silica surfaces (20, 29). We thus demonstrate a rapid, simple, optical-snapshot-based approach to the characterization of the electrical charge of an arbitrary 2D material in contact with a fluid. Once characterized, a surface of known $\phi_{s}$—in this case silica—may be used either to characterize the effective charge, $q_{eff}$, of a new probe molecule (Fig. 2*D* and *SI Appendix*, Fig. S9), or, in conjunction with a well-known probe molecule, to measure the properties of a new material coated on the opposite surface (Fig. 3).

### Electrostatic Imaging and Surface Characterization of a Variety of Thin-Film Materials.

Next, in order to demonstrate the generality of the method, we performed electrostatic imaging of thin films composed of a wide range of positively and negatively charged materials such as Al_2_O_3_, HfO_2_, and TiO_2_ representing inorganic oxides, as well as positively and negatively charged polymer and polyelectrolyte coatings such as polyethyleneimine (PEI), polystyrene sulfonate (PSS), and polymethyl methacrylate (PMMA) (Fig. 3 *A–G*). The results are summarized in Fig. 3*H*. In particular, for measurements at pH 6, we note large magnitudes of surface potential for the negatively charged polymer films PSS ($\psi_{s}=-4.2\;\pm \;1.1$; $\sigma =-0.03\;\pm \;0.02\;\;/{\mathrm{nm}}^{2}$) and PMMA ($\psi_{s}=-3.4\pm 0.4$; $\sigma =-0.02\pm 0.004\;e/{\mathrm{nm}}^{2}$), and slightly lower magnitudes of potential in general for both inorganic oxide films of Al_2_O_3_ ($\psi_{s}=1.7\pm 0.8$; $\sigma =0.009\;\pm \;0.006\;e/{\mathrm{nm}}^{2}$) at pH 4.5 and TiO_2_ ($\psi_{s}=-2.9\pm \;0.8$; $\sigma =-0.02\pm 0.01\;e/{\mathrm{nm}}^{2}$) at pH 9, and the positively charged polyelectrolyte PEI ($\psi_{s}=2\pm 0.7$; $\sigma =0.010\pm 0.005\;e/{\mathrm{nm}}^{2}$) at pH 4.5. We attribute the relatively high magnitude charge on our anionic polymeric films to the strongly acidic nature of the ionizable groups, reflected in the relatively low value of p*K*_a_ ≈ 2 for sulfonate groups in PSS and p*K*_a_ ≈ 5 for carboxyl groups in PMMA (48, 49). Furthermore, metal oxides generally display amphoteric behavior with isoelectric points between 3 and 8 such that the material is positively charged when pH < pI and negative when pH > pI. The range of probe molecules available renders our method well suited to measurements on both positive and negatively charged surfaces. For example, measurements at various pH on a thin film of HfO_2_ reveal values of $\psi_{s}=-3.5\pm 0.7$ ($\sigma =-0.019\;\pm \;0.007\;e/{\mathrm{nm}}^{2}$) at pH ≈ 9, and $\psi_{s}=2.0\;\pm \;0.6$ ($\sigma =0.008\;\pm \;0.003\;\;/{\mathrm{nm}}^{2}$) at pH ≈ 4.5, permitting us to place the pI of HfO_2_ at ≈7 and that of TiO_2_ at ≈6 (Fig. 3*H*), both in good agreement with reported zeta potentials measured for particulate matter in suspension (50, 51). Note that when the solution pH is very close to the isoelectric point of the surface ($\psi_{s}\approx 0$), weakly charged probe molecules (${|q}_{eff}|=1e$) may adsorb to the surface. We do not quantify the surface potential from such experiments as the surface is obviously modified by the adsorbing molecules (*SI Appendix*, Fig. S14). We do however have the option of switching to a more highly charged probe molecule ($q_{eff}=-2$ to -3e, or higher if needed), which permits us to reliably probe surface electrical properties in situations where a weakly charged probe molecule shows evidence of surface adsorption. This point is described in further detail in SI (*SI Appendix,* Figs. S9, S10, and S11).

**Fig. 4.**
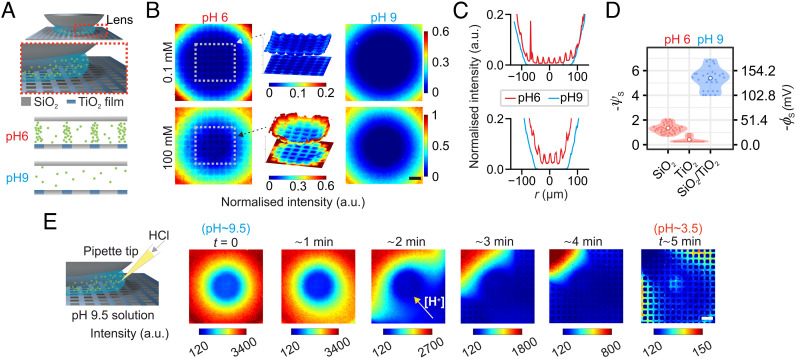
Spatiotemporal imaging of electrical charge modulation on a chemically heterogeneous surface. (*A*) Schematic representation of the variable-height gap where the SiO_2_ substrate is patterned with a square grid of TiO_2_ lines (*Top*). Schematic depiction of the system cross-section depicting distributions of probe molecule in the gap, based on surface potential measurements for TiO_2_ and SiO_2_ at pH ≈ 6 and pH ≈ 9 from Fig. 3 (*Bottom*). (*B*) Fluorescence images of the probe molecule (ATTO 542c, $q_{str}=-3e$) distribution at pH ≈ 6 and pH ≈ 9 in low (c=0.1 mM NaCl) and high ionic strength (c=100 mM NaCl) solutions. A 3D surface plot of spatial intensity for the center region of each image at pH ≈ 6 highlights the contrast due to the different surface potentials of SiO_2_ and TiO_2_. The scale bar corresponds to 40 μm and all images in (*B*) have the same scale. (*C*) Intensity profiles along the center lines of the images in (*B*). (*D*) Values of electrical surface potential in regions corresponding to different surface materials on the patterned substrate, inferred from intensity profiles shown in (*C*) at pH ≈ 6 and 9 at low ionic strength. White symbols depict the mean value of potential inferred for each material. (*E*) Temporal measurement of the variation in electrostatic contrast for the system in (*A*), induced by the addition of concentrated HCl. A proton gradient emanating from the location of the pH perturbation triggers a spatiotemporal change in solution pH such that the initial pH of the system is ≈9.5 at t=0 and decreases to ≈3.5 at t≈5 min. The scale bar corresponds to 40 μm and all images in (*E*) have the same scale.

Having established the basic framework of our opto-electrostatic-imaging technique using nominally homogeneous thin films, we then focused on the examination of surfaces carrying a heterogeneous surface charge distribution. We constructed a model heterogeneous surface by coating glass coverslips with ≈10-nm-thick TiO_2_ film, and using optical lithography and H_3_PO_4_ etching to pattern a periodic square grid with a 10-μm pitch as shown in Fig. 4*A* and *SI Appendix*, Fig. S12. In order to ensure substantial optical contrast in the measurement (as shown in the simulations described in *SI Appendix*, Figs. S10 and S11), we use a highly charged probe molecule, ATTO 542c, expected to carry a nominal net electrical charge of $q_{eff}=-3e$ (chemical structure not available). We performed electrostatic imaging of the patterned surfaces in contact with electrolytes at two different pH values. At pH ≈ 6 which is close to the pI for TiO_2_ and much larger than that of SiO_2_ (pI < 3), homogeneous thin film measurements (Fig. 3*H*) show that TiO_2_ carries little charge ($\psi_{s}=-0.7\;\pm \;0.6$; $\sigma =-0.001\;\pm \;0.0004\;e/{\mathrm{nm}}^{2}$) whereas SiO_2_ is expected to be substantially charged ($\psi_{s}=-3.1\;\pm \;0.3$;  $\sigma =-0.019\;\pm \;0.005\;e/{\mathrm{nm}}^{2}$). At pH ≈ 9, however, we found that both materials can be expected to carry substantial and comparable amounts of charge ($\left|\psi_{s}\right|\approx 3$; $\left|\sigma \right|\approx 0.02\;e/{\mathrm{nm}}^{2}$). Fig. 4*B* displays optical snapshots of the surface electrostatics for the chemically patterned surfaces at both low (c≈0.01 mM) and high (c≈100 mM) NaCl concentrations using ATTO 542 c (-3e) as the probe molecule. For low salt concentration at pH 6, we observed a marked contrast in optical intensity between the glass (dark square regions) and TiO_2_ regions (bright grid lines) of the coverslip, reflecting a lower magnitude of $\psi_{s}$ in the TiO_2_ regions as expected from the homogeneous thin film measurements. A cross section through the grid emphasizes the electrostatically generated optical contrast due to the grid pattern that appears superimposed on the macroscopic exclusion zone feature created by the lens curvature (Fig. 4*C*, red curve). On the other hand, at pH ≈ 9, the optical intensity modulation due to the material heterogeneity largely vanishes (Fig. 4*C*, blue curve) and only a featureless exclusion region remains, reminiscent of a homogenous thin film measurement. At high salt concentrations (c≈100 mM NaCl), for both pH ≈ 6 and pH ≈ 9, we observe a reduction in the radius of the exclusion zone due to screening as expected (Fig. 4 *B* and *C*, *Bottom* *row*). Here again, we note the visually apparent grid-induced modulation of spatial electrostatic contrast at pH ≈ 6, and an absence of such contrast at pH ≈ 9. The spatial modulation in optical contrast appears to arise solely from the chemical response of the ionizable surface groups of both materials. We note that the lens-based confinement system yields an area-averaged measure of the surface potential and charge density for homogeneous or periodically heterogeneous surfaces (Fig. 4*D*).

### Spatiotemporal Monitoring of Surface Chemical Reactions.

Using this system, we further demonstrate the ability to optically image dynamic changes in charge and chemistry at the solid–liquid interface. In order to do so we begin with an alkaline solution (pH ≈ 9) in the gap and trigger a change in the pH by adding HCl, giving a final pH of ≈ 3.5. Immediately following the introduction of acid, we image the system using exposure times of $t_{exp}=\;10$ ms. As protons diffuse through the system the surface groups can respond to the local H_3_O^+^ concentration in solution by changing their protonation state. This in turn can alter the local surface electrical potential, directly impacting the local fluorescent probe concentration in solution and the measured local optical intensity (Fig. 4*E* and Movie S1). At t=0, we observe no optical contrast due to chemical heterogeneity at the surface. As time progresses and the pH in the gap drops due to diffusion of acid, we gradually observe the appearance of a grid-induced spatial modulation of optical contrast since SiO_2_ retains substantially more surface charge than TiO_2_ at low pH. We thus achieve optical observation of changes in surface chemistry induced by the diffusion of protons through the field of view. The timescale of the propagation of about 5 min is commensurate with the diffusion time of the hydronium ion over a distance of approximately 2.5 mm, which is overall a rather slow process (52). However, the time resolution of the acquisition, $t_{\exp}=10$ ms, would support observation of much faster chemical and physical dynamics using this technique.

### A Scanning Probe System to Image Chemically Heterogeneous Surfaces Over Large Areas.

**Fig. 5.**
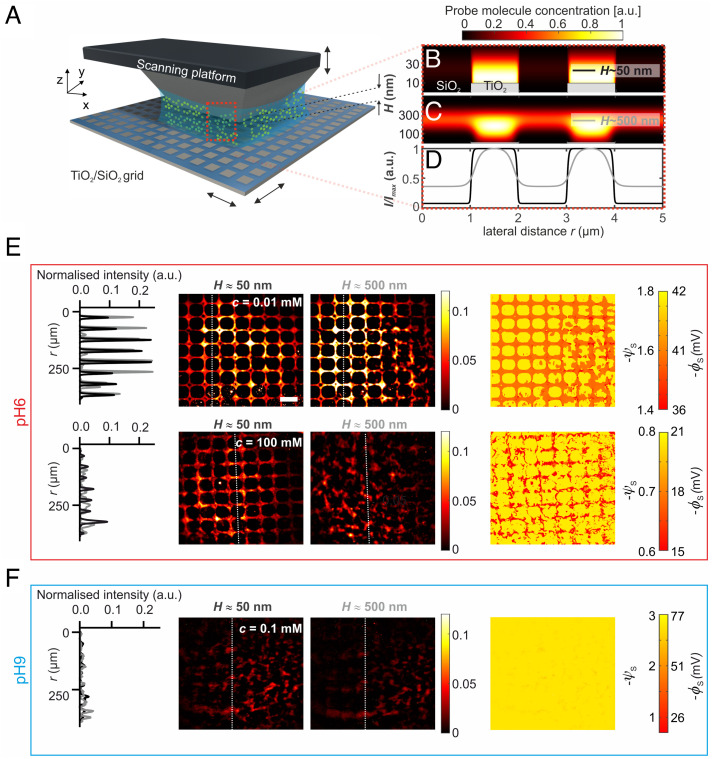
Imaging of spatial electrical surface charge distribution on a chemically heterogeneous surface. (*A*) Schematic depiction of a scanning-probe setup for electrostatic contrast measurements on heterogeneous substrates (not to scale). *H* denotes the height of the gap between the scanning platform and the test surface. (*B*,*C*) Calculations of the spatial distribution of probe molecules with a charge of $q_{str}=-3e$ (ATTO 542c) using Eq. **2** for gap heights of *H* ≈ 50 nm (*B*) and *H* ≈ 500 nm (*C*) in low ionic strength solution (*c *= 0.01 mM) at pH 6. (*D*) Calculated intensity profiles for (*B*) in black and (*C*) in gray based on Eq. **3**. The TiO_2_ regions of the substrate appear brighter because they carry a lower surface charge density than SiO_2_ (Fig. 3*H*). (*E*) *Middle Panels:* images of fluorescence intensity distributions at two heights of the scanning platform (*H* = 50 nm and 500 nm), in low and high ionic strength solutions at pH ≈ 6. *Left*: plots of intensity profiles averaged over the dotted lines in the images. Spatial intensity modulation is clearly visible at pH 6 where there is a large disparity in surface charge density between TiO_2_ and SiO_2_. *Right*: Spatial distribution of the electrical surface potential on the substrate inferred from the measured fluorescent intensity distribution at two heights for low and high ionic strength at pH ≈ 6 (See *SI Appendix*, Fig. S13 for corresponding surface charge density maps). Speckles in the presented images arise from the use of a diffuser in the optical setup, which can at present influence the uncertainty on the inferred surface potential by a small amount estimated at ±5 mV. (*F*) Similar information as in (*E*) for experiments performed at pH 9. Spatial variation in intensity vanishes due to the similarity of electrical surface properties of the two materials under the experimental conditions. The scale bar corresponds to 20 μm and is the same for all images.

Finally, we further extend the demonstrated opto-electrostatic measurement principle to a scanning probe system (Fig. 5). Here, the scanning probe or platform is a flat-topped pyramidal silicon/SiO_2_ structure of lateral dimension d≈300 µm and height of 50–100 µm (Fig. 5*A* and *Methods*). We monitor the height of the platform surface above the scanned substrate surface with nanometric control using white light interferometry (*SI Appendix* and *Materials and Methods*). We imaged the SiO_2_/TiO_2_ patterned substrates by laterally scanning the substrate with respect to the Si/SiO_2_ platform surface positioned at a height, H, above the substrate. Similar to the measurements in the lens-based system, we observed strong optical contrast between the TiO_2_ and SiO_2_ regions at pH ≈ 6 (Fig. 5*E* and Movies S2 and S3). At low salt concentrations (c≈0.01 mM NaCl; $\kappa^{-1}\approx 100$ nm), we observe a bright signal from the regions corresponding to the TiO_2_ grid lines at gap heights of H≈50 nm (SNR ≈ 2.5), with a progressive increase in contrast with increasing heights up to ≈500 nm (Fig. 5*E*). At even larger gap heights, the contrast decreases progressively and finally vanishes due to the reduction in the impact of interfacial electrostatics on the measured intensities relative to the contribution from the probe molecule in bulk solution (Movie S2 and *SI Appendix*, Fig. S11*B*). At higher salt concentrations, ca. 100 mM ($\kappa^{-1}\approx 1$ nm), we noted that the electrostatic contrast was large for gap heights H<50 nm and disappeared entirely for H>50 nm as expected (Fig. 5*E * and Movie S3). The observations are all in line with the general expectation that the SNR of the optical contrast is maximal at κH≈3  (*SI Appendix*). Yet again, similar to the observations using the lens as a probe surface, we detected no electrostatic contrast between the SiO_2_ and TiO_2_ regions at pH ≈ 9 (Fig. 5*F* and Movie S4), regardless of salt concentration, consistent with expectations based on homogenous thin film measurements (Fig. 3*H*) and calculated images (*SI Appendix*, Fig. S11*B*). In the lens-based confinement system, where the height profile is built-in, we obtain fluorescence intensity values at locations on the substrate that each correspond to a particular value of the gap height. Unlike the lens-based system, an axially scannable flat surface system permits measurements of the same spatial location on the substrate at multiple different heights. This permits multiplicative and additive factors in the measured intensities to be eliminated from the analysis. As a result, we are able to construct spatial surface potential and charge density maps as shown in Fig. 5 *E* and *F* and *SI Appendix,* Fig. S13.

We further performed calculations to determine the minimum feature size of a weakly charged material (such as TiO_2_) that could be reliably measured against a highly charged background, similar to silica using this technique (*SI Appendix*, Fig. S10*A*). Our analysis shows that the electrostatic imaging approach can detect feature sizes on the order of ≈80 nm at a salt concentration of 0.1 mM NaCl ($\kappa^{-1}\approx$30 nm, H=110 nm). The electrical contrast detected in this case corresponds to surface electrical potential differences of ${\Delta \psi }_{s}\approx 0.5$ (${\Delta \phi }_{s}\approx 12$ mV) corresponding to a difference of surface charge of $\Delta \sigma \approx 5\;\times 10^{-3}\;e/{\mathrm{nm}}^{2}$ in 0.1 mM NaCl solution (*SI Appendix*, Fig. S11 *C–E*). In general, the minimal detectable feature size corresponds to about twice the Debye length in a system with $H\approx 2\kappa^{-1}$. This further implies that the smallest discernible feature size, s, can be tuned by varying the salt concentration of the solution, i.e., $s\propto \kappa^{-1}\propto 1/\sqrt{c}$.

## Discussion

An understanding of interfacial processes is vital to the successful control and engineering of a range of chemical processes, but hinges on the availability of versatile measurement techniques that provide a sensitive readout of interfacial chemical and electrical states. Methods to measure forces and interactions at the solid–liquid interface have been developed at the macroscopic scale, e.g., the Surface Force Balance and at the micro- and nanoscopic scale using atomic force microscopy (AFM) (53, 54). The latter family of methods has been further extended to probe the interaction of functional groups at the surface with highly specialized chemical force microscopy probes (55, 56), but measurements of the interaction between a molecule in the fluid phase and a surface remain challenging.

Our study demonstrates a measurement of the interaction between a molecule and the solid–liquid interface using a simple thermodynamic principle introduced in previous work (27, 29). In our approach, a light-emitting molecule diffusing in solution acts as a scanning probe driven by thermal energy. The molecular probe engages in a three-dimensional random walk and explores interaction energy landscape created by surfaces in the vicinity. Optical observation of a number of such thermally driven probes in parallel permits us to measure the local interaction of the molecule with the surface of interest and enables inferences on the properties of the surface in a single, static optical snapshot. Use of an electrically charged probe molecule in these experiments has facilitated large area electrical, and therefore chemical, characterization at submicron (spatial) and millisecond (temporal) resolution of a variety of surface materials and thin films immersed in a solvent. Importantly, in conjunction with appropriate physical models of the underlying interactions and the optical response of the confined probe molecule, the acquired images further permit quantitative mapping of the ionization constants of chemical groups distributed across an inhomogeneous surface. The measurement approach is not limited to thin films deposited on flat, transparent substrates but may be adapted to opaque, nonflat surfaces and materials where the spatial optical intensity distribution can be measured by images acquired through a transparent probe surface of known surface properties. In the characterization of electrical charge of surface materials, we expect this approach to have a reach and relevance comparable with the now ubiquitous electrokinetic (zeta potential) measurement technique for particulate dispersions, with the important added dimension of high spatiotemporal measurement resolution, enabling charge-state alterations due to surface chemical reactions, e.g., to be monitored in real time. Future measurements will extend this concept beyond electrostatic interactions to the measurement of molecule-surface interactions in solution in a more general sense.

## Materials and Methods

### Wide-Field Fluorescence Microscopy.

The variable height gap between the lens and coverglass surface is created by placing an uncoated 25 mm focal length planoconvex N-BK7 lens (LA1951, Thorlabs) on top of a No. 1.5 borosilicate coverglass 170 µm thick (22 × 22 mm^2^, Menzel) (Fig. 1*A*), which itself rests on a 12-mm-diameter circular aperture in an aluminum sample holder machined in-house. A 45-µL droplet of a solution containing the charged, light-emitting probe at a concentration between 100 and 200 nM was pipetted into the gap for experiments.

The wide-field microscope (shown schematically in Fig. 1*A*) consists of a diode-pumped solid-state (DPSS) 532-nm laser beam which is passed through an active diffuser (Optotune), expanded with a 20× telescope, reflected off a dichroic mirror (ZT488/532rpc, Chroma), and then focused onto the back focal plane of a 60×, 1.25 numerical aperture (NA) objective (Olympus) to generate wide-field illumination for dye excitation. Images of the dye distribution were captured with a scientific complementary metal oxide semiconductor camera (BSI Prime, Photometrics). The effective pixel size in the imaging plane was measured to be ≈127 ± 5 nm. A nonpolarizing beam splitter cube (Thorlabs) was used to redirect and image the reflected laser excitation from the sample onto a charge-coupled device (CCD) camera (Thorlabs). The resulting Newton’s rings interference pattern was analyzed to extract precise height information in the lens–coverglass gap (*SI Appendix*, Fig. S1*B*). The captured fluorescence images ($I_{0}$) were processed to remove background signal ($I_{bg}$) and to correct for the laser Gaussian illumination profile, yielding an intensity distribution that may be written as $I_{c}=\left(I_{0}-I_{bg}\right)/I_{ill}$, as shown in *SI Appendix*, Fig. S1*A*. The images were analyzed further as described in *SI Appendix*, Sections 1–5 in order to extract spatially dependent electrical and chemical information on the surfaces under test.

### Surface Cleaning Procedure.

All coverglasses and lenses were initially cleaned by sonication for 20 min in acetone (Sigma-Aldrich), followed by sonication in isopropyl alcohol (IPA, Sigma-Aldrich). After rinsing for 2 mins with DI water, substrates were cleaned in piranha solution (H_2_O_2_: H_2_SO_4_ 50:50%) to remove all organic residues and then rinsed and immersed in DI water for >25 mins prior to imaging.

### Electrolyte Solution Preparation.

In order to prepare a solution of fluorescent probe molecules at pH ≈ 6, we dissolved 10 µL of a 10 µM stock of fluorescent dye in 990 µL of solutions of increasing salt concentration (DI water, 0.1 mM, 1 mM, and 100 mM NaCl) to obtain a ≈100 nM dye concentration at pH ≈ 6. The dyes used for the negative probe experiments (Figs. 2, 3 *A–D*, 4, and 5) were ATTO 532NHS, ATTO 532c, and ATTO 542c (ATTO-TEC Gmbh). Experiments at pH ≈ 9 were performed by adding 10 µL of 100 mM TRIS (Sigma-Aldrich) to 980 µL of the electrolyte, followed by the addition of dye solution as described above. pH ≈ 4.5 solutions for the positive probe experiments (Fig. 3 *E–G*) were prepared by adding 10 µL of 10 mM HCl and 10 µL of 10 µM stock of ATTO Rhodamine 6G amine (ATTO-TEC Gmbh) to 980 µL of the NaCl solutions. To prepare the electrolyte solutions with ssDNA as a probe, eight base ssDNA (5′TAG AAC TA3′, Microsynth) and nine base ssDNA (5′TAG AAC TAG3′, Microsynth) doubly labeled with ATTO 532NHS were diluted to ≈10 µM concentration. Ten microliters of 10 µM ssDNA stock solution was added to 10 µL of 100 mM TRIS (Sigma-Aldrich) and 980 µL of the stock NaCl solutions in order to make DNA samples at pH 9. The conductivity and pH of the prepared solutions were measured prior to the experiments using small-volume pH and conductivity meters (LAQUA-TWIN, Horiba).

### Polyelectrolyte and Polymer Thin Film Coatings.

PEI coatings (Fig. 3*E*) were obtained by immersing clean coverglasses (prepared as described above) and lenses in 1% weight-to-volume solutions of branched PEI (mol. wt. 750,000) for 45 min, and subsequently rinsing for 2 min with DI water. The coated lenses and coverglasses were allowed to dry in air for 1 h before imaging. PSS coatings (Fig. 3*A*) were obtained by repeating the aforementioned coating procedure on freshly coated PEI substrates. Here, PEI-coated surfaces were immersed in 1% weight per volume solutions of PSS (mol. wt. 70,000) in water. PMMA (Fig. 3*B*) films were prepared by spin-coating PMMA 950k in anisole solvent (6% weight per volume) onto the coverglasses at 3,000 rpm, followed by baking the substrate on a hotplate at 180°C for 3 min.

### Atomic Layer Deposition (ALD) of HfO_2_ and Al_2_O_3_ on Coverglasses.

A uniform coating of coverglasses with two different oxides, namely hafnia (HfO_2_) (Fig. 3 *C* and *F*) and alumina (Al_2_O_3_), (Fig. 3*G*), was achieved using ALD. Coverglasses, cleaned as described previously, were coated with thin oxide layers of thickness between 10 and 20 nm using a thermal ALD deposition system (Veeco Savannah).

### Preparation of Patterned TiO_2_ Thin Films on Borosilicate Coverglasses.

A layer of TiO_2_ was deposited on 170-µm-thick borosilicate coverglasses using electron beam sputtering, with resulting thicknesses ranging between 10 and 20 nm. Coverglasses uniformly coated with TiO_2_ were used for the surface potential measurements reported in Fig. 3 *F* and *H*. Coverglasses with a uniform layer of TiO_2_ were further spatially patterned for experiments shown in Figs. 4 and 5, and *SI Appendix*, Fig. S12 by first coating with 10-nm chrome and 30-nm gold films, deposited by thermal evaporation. These metalized TiO_2_ surfaces were subsequently coated with a 2-µm-thick layer of negative resist (AZ2020 nLOF, MicroChemicals GmbH) and patterned using photolithography to form a grid pattern defined in the resist layer. Following the photolithography step, the exposed regions of the Cr/Au layers were removed by wet etching. The resulting patterned substrates consisting of alternating TiO_2_/Cr/Au/resist and uncovered TiO_2_ regions were etched using H_2_SO_4_ at 95°C in order to selectively remove the uncovered regions of TiO_2_. The resist was then stripped by heating the coverglasses in dimethyl sulfoxide for 12h at 70°C, followed by 5 min sonication in acetone and 5 min sonication in IPA. Finally, the metal films were removed by wet etching in Au and Cr etching solutions. The patterned surfaces were then characterized using AFM (Bruker) (*SI Appendix*, Fig. S12).

### Scanning-Probe-Based Wide-Field Fluorescence Microscopy.

The scanning-probe-based system shown in Fig. 5*A* was mounted on a wide-field microscope constructed along lines similar to the setup depicted in Fig. 1*A*, albeit with different components. A DPSS 532-nm laser beam was passed through an active diffuser (Optotune), expanded with a 20× telescope, reflected off a dichroic mirror (ZT488/532rpc, Chroma), and then focused onto the back focal plane of a 40×, 0.75 NA air objective (Nikon). Images were relayed onto an electron-multiplying CCD camera (Andor Luca-R) using a 100-mm focal length tube lens (Thorlabs).

### Interferometric Height Determination in Scanning-Probe-Based System.

In the scanning-probe-based system shown in Fig. 5, the probe surface (thermally grown SiO_2_) is effectively flat. Parallel alignment of the two substrates with respect to each other was achieved through fine angular control with linear piezo actuators (Newport Picomotor) ensuring a uniform height throughout the gap in the imaged field of view. In this case, the illumination wavelength is varied using a white light source and a variable bandpass filter (bandwidth 400–750 nm) that is sequentially scanned across the illumination spectrum from wavelengths from 420 nm to 720 nm in 10-nm steps. The reflected intensity from the sample is then imaged onto a CCD camera (Thorlabs). The collected intensity profiles are corrected for background and illumination profile (using a procedure similar to that presented in *SI Appendix*, Fig. S1 and described in *SI Appendix*) and then fit using the transfer matrix method described for the interferometry in the lens-based system (*SI Appendix*, Eq. **S1**). The multilayer stack in the scanning-probe based system is glass ($n_{1}=1.515$), water ($n_{2}=1.330$), glass ($n_{3}=1.515$), and silicon ($n_{4}\approx 4.15$). The precision of the gap height determination is ±5 nm, for a height range between 50 nm and 3 µm. The height, H, between the probe platform and the surface of interest was varied using precision micrometer screws and a three-axis NanoMax stage (Thorlabs).

### Fourier Filtering of Scanning-Probe System Images.

The background correction of the images from the scanning-probe system was performed differently to the lens-based system. Rather than the subtraction and division procedure described in *SI Appendix*, Fig. S1*A* for the lens–coverglass system, we used low-pass filtering in Fourier space. We found that a low-pass filter on spatial frequencies was effective at removing background intensity arising from interference patterns due to the reflection from the silicon surface and the small relative tilt between the two facing surfaces of the scanning cantilever. Removing the dc background (0-frequency component of the Fourier space image), resulted in a high-contrast image as shown in Fig. 5*E*. Filtering was implemented by taking the Fourier transform of a 50-frame image stack, removing a circular region from the center of the transform, and then taking the inverse Fourier transform of the filtered intermediate image to obtain a background-corrected image stack. The results of the background correction are shown in the images in Fig. 5 *E* and *F*.

## Supplementary Material

Appendix 01 (PDF)

Movie S1. Time lapse of the moving H+ wave across the field of view of the variable-height gap presented in Fig. 4*D*. The solution changes from pH ≈9.6 to 3.5 as the protons diffuse across the system, altering the electrical charge on the surface and rendering local chemical heterogeneity visible in the form of optical contrast.

Movie S2. Height sweep of the flat scanning probe from Fig. 5*E*, using a 0.01 mM NaCl solution at pH6. Due to a difference in the electrostatic surface potential between the materials at pH 6, a strong contrast is observed between the TiO_2_ areas (lines surrounding the squares) and the SiO_2_ (material inside the squares). At large heights (*H*>500 nm), the contrast disappears as the collected fluorescent emission is dominated by dye molecules in the “bulk solution” in the gap that is relatively insensitive to the surface electrical potential.

Movie S3. Height sweep of the flat scanning probe from Fig. 5*E*, for an experiment in 100 mM NaCl solution at pH6. Due to differences in the electrostatic surface potential between the materials at pH6, a strong contrast is observed between the TiO_2_ areas (lines surrounding the squares) and the SiO_2_ (material inside the squares). The contrast is only visible up to small heights (*H*>500 nm), as the length scale of the electrostatic interactions is shorter and given by the Debye length, approximately 1 nm in this case.

Movie S4. Height sweep of the flat scanning probe from Fig. 5*F*, using a 0.1 mM NaCl solution at pH9. No contrast is observed between the TiO_2_ areas (lines surrounding the squares) and the SiO_2_ (material inside the squares), as the electrostatic surface potentials of both materials are similar under these conditions $\left(|\psi_{\text{s},{\text{SiO}}_{\text{z}}}|\approx |\psi_{\text{s},{\text{SiO}}_{\text{z}}}|\approx 3\right)$

## Data Availability

Experimental data for silica measurements are hosted on the Zenodo public repository at https://doi.org/10.5281/zenodo.7111999 (57). Data for the characterization of polymers (https://doi.org/10.5281/zenodo.7111592) (58), metal oxides (https://zenodo.org/record/7111790) (59) as well as TiO_2_/SiO_2_ patterns (https://doi.org/10.5281/zenodo.7114460) (60) were made available on Zenodo. Codes for the experimental data analysis and simulations are accessible on Zenodo at https://doi.org/10.5281/zenodo.7110758 (61). All other data are included in the article and/or *SI Appendix*
