# Supplementary material for: Wide-field optical imaging of electrical charge and chemical reactions at the solid–liquid interface
Source: Proc Natl Acad Sci U S A. 2022 Dec 2;119(49):e2209955119. doi: 10.1073/pnas.2209955119 (PMC9894211; doi:10.1073/pnas.2209955119)
Supplement: Supplementary file 1 — Appendix 01 (PDF) [file pnas.2209955119.sapp.pdf]

Supplementary Information for

Wide-field optical imaging of electrical charge and chemical reactions at the solid-liquid interface

Sushanta Mahanta[†1], Pedro Vallejo-Ramirez[†1], Narain Karedla[†1], Pawel Puczkarski[1] & Madhavi Krishnan*[1,2]

[†]these authors contributed equally
*corresponding author

Corresponding Author information: Madhavi Krishnan
Email: madhavi.krishnan@chem.ox.ac.uk

Author affiliations:

[1]Physical and Theoretical Chemistry Laboratory, Department of Chemistry, University of Oxford, South Parks Road, Oxford OX1 3QZ, United Kingdom

[2]The Kavli Institute for Nanoscience Discovery, Sherrington Road, Oxford OX1 3QU, United Kingdom

This PDF file includes:

Supplementary text
Figures S1 to S14
Legends for Movies S1 to S4
SI References

Other supplementary materials for this manuscript include the following:

Movies S1 to S4

Supplementary Information Text

1. Interferometry-based height measurement in the lens-coverglass system

The height in the gap created between lens and coverglass was measured using the interferometric pattern (Newton's rings) created by reflection of excitation laser light from the two flanking surfaces. This pattern was recorded on a CCD camera (Thorlabs) as shown in Fig. 1*A*. First, the centre of the interference pattern was found by binarizing the image using increasing grey level thresholds, and subsequently finding the centre of mass of the radially symmetric binarized pattern for each increasing threshold value, as previously described by Morrin *et al*. (Fig. S1*B*)(1). The true centre of mass of the image is estimated as the mean value of the estimated centre of mass for all increasing thresholds. The centre coordinates are then used to take a radial average over the image to generate a radial intensity trace, $\tilde{I}(r)$, which is typically a trace from the centre ($r = 0$) to the edge of the field of view ($r = 130$ µm) containing 3 intensity minima and 3 maxima, (red and green symbols overlaid on trace in Fig. S1*B*).

The variable-height gap in our measurements consists of a well-defined multi-layer geometry, glass/water/glass (Fig. 1-2) and glass/thin film/water/glass (Fig. 3-4). We used the Fresnel equations to calculate the reflection and transmission coefficients at each interface. Since our system is illuminated with collimated light, we assumed normal incidence for these calculations. The optical transfer matrix method was used to calculate the reflected intensity with respect to the height H of the water-filled gap.(2) Fig. S1*B* displays minima and maxima for a calculated interference pattern as a function of height H of the system. The calculated intensity from each minimum to the following maximum can be mapped to the measured intensity trace in a piecewise fashion in order to determine the height as a function of radial location in the system. If D_{ij} is defined as the transfer matrix between two materials with refractive index n_i and n_j and P_k is the propagation matrix within the material whose index is n_k, then the total transfer matrix can be written as $M = D_{12}P_2D_{21}$ where n_1 is the refractive index for medium 1 (glass), and n_2 is the refractive index for medium 2 (water). To extract the intensity of the reflected light, we use the solution for the reflection coefficient $R = \frac{M_{21}}{M_{11}}$, where the indices identify the element in the 2×2 propagation matrix. The transmission coefficient T can be obtained from the following equation $T^2 = 1 - R^2$.

The final solution for R is given by:

$$R = \frac{\frac{e^{-\frac{2iL_2n_2\pi}{\lambda}}(n_1+n_2)(n_2-n_3)}{4n_1n_2} + \frac{e^{\frac{2iL_2n_2\pi}{\lambda}}(n_1-n_2)(n_2+n_3)}{4n_1n_2}}{\frac{e^{-\frac{2iL_2n_2\pi}{\lambda}}(n_1-n_2)(n_2-n_3)}{4n_1n_2} + \frac{e^{\frac{2iL_2n_2\pi}{\lambda}}(n_1+n_2)(n_2+n_3)}{4n_1n_2}} \quad \text{(S1)}$$

where $n_1 = n_3 = 1.515$, $n_2 = 1.33$, $\lambda = 532$ nm, and $L_2 = H(r)$, for $H(r)$ ranging from 0 - 500 nm in the gap. The results of the piecewise fitting of the calculated versus the measured intensity as a function of height are shown in Fig. S1*D-E*.

The optical transfer matrix method makes it straightforward to add the refractive indices of thin layers of materials, for example thin oxide films such as TiO_2, HfO_2 or Al_2O_3 (Fig. 3-5), in the gap, enabling accurate height measurements in the presence of deposited thin films on the silica substrate. This is achieved by using a general form of the transfer matrix for an *m*-layer optical stack

$$M = D_{12} \sum_{i=2}^{m-1} P_i D_{i,i+1} \quad \text{(S2)}$$

with the transmission matrix D_{ij} and propagation matrix P_i, which we can write as

$$D_{ij} = \begin{pmatrix} 1 & \frac{n_i - n_j}{n_i + n_j} \\ \frac{n_i - n_j}{n_i + n_j} & 1 \end{pmatrix} \frac{(n_i + n_j)}{2n_i}, \quad P_i = \begin{pmatrix} e^{\frac{i2\pi L n_i}{\lambda}} & 0 \\ 0 & e^{-\frac{i2\pi L n_i}{\lambda}} \end{pmatrix} \quad \text{(S3)}$$

Finally, the reflection coefficient is determined in the same way as described above:

$$R = \frac{M_{21}}{M_{11}} \quad \text{(S4)}$$

We generally only consider two interfaces (a glass/water and water/silica) interface in our experiments. We include an additional interface when the experiments include metal oxide films (Fig. 3*C-D*, $n_{\mathrm{TiO_2}} \approx 2.8$, $n_{\mathrm{HfO_2}} \approx 1.9$, $n_{\mathrm{Al_2O_3}} \approx 1.7$). We do not consider additional interfaces in experiments involving polymer coatings, as the films are reported to be extremely thin and the refractive indices of polymers are close to glass.(3, 4)

2. Poisson-Boltzmann numerical model for surface potential and surface charge density determination

The values of surface electrical potential and charge density reported in this work were obtained by matching the experimentally measured radial intensity distributions of the light-emitting probes ($I(r)$) with theoretical intensity profiles calculated using the Poisson-Boltzmann (PB) equation for the variable height gap. In this section, we describe the analysis procedure using the PB equation to determine the theoretically expected probe molecule concentration profiles in the system. Importantly we also use optical corrections to account for fluorescent dye emission coupling into

the substrate surfaces in order to arrive at a theoretical $I(r)$ profile under a given set of experimental conditions.

The PB equation can be expressed in dimensionless form as:

$$\Delta\psi = \kappa^2 \sinh(\psi) \quad \text{(S5)}$$

where $\psi = \frac{e\phi}{k_B T}$ is the dimensionless electrical potential ϕ and $\kappa^{-1} = \sqrt{\frac{\varepsilon_0 \varepsilon_m k_B T}{2 N_A c e^2}}$ is the Debye length. Here ε_0 is the vacuum permittivity, ε_m the permittivity of the medium, k_B is the Boltzmann constant, T is the temperature, N_A is the Avogadro number, c is the salt concentration in solution, and e is the elementary charge. We specify the boundary conditions of the problem, which can be either a Dirichlet boundary condition of fixed potential ψ_s at the surfaces, or a Neumann boundary condition which entails a constant electric field normal to the surface. We apply a Neumann boundary condition at the surfaces, which can be expressed as

$$\boldsymbol{n} \cdot \nabla\psi = \frac{\sigma e}{\varepsilon_0 \varepsilon_m k_B T} \quad \text{(S6)}$$

where $\boldsymbol{n}$ is the surface normal pointing into the gap. Eq. (S6) corresponds to a constant surface charge density, σ, at the bounding surfaces.

Radial symmetry and the short length scales of our experimental setup facilitate rapid computation of the electrical potential distribution ($\psi(r, z)$) in the gap, described by the NLPB equation. The gap created by the lens in the experiment is cylindrically symmetric around the contact region with the bottom substrate, allowing us to reduce the dimension of the simulation to a 2D axisymmetric model (Fig. S3*A*). We can further reduce the dimensionality of the system under the assumption that the curvature of the lens is very small. This assumption is valid because within the radial distance covered in the field of view (0 µm < r < 110 µm), we measured a gap height H between 0 and 500 nm. Hence, the solution for the electrical potential distribution in the gap can be approximated by solving the NLPB in 1D for each height H thus enabling us to reconstruct the solution for the entire axisymmetric geometry based on a series of 1-D solutions at variable H (Fig. S3*B*). We further illustrate this reduction in dimensionality in Fig. S3, where both the 2D axisymmetric and 1D potential distributions were solved in COMSOL and found to be identical within numerical precision (Fig. S3*C*-*D*).

Having obtained the potential distribution $\psi(r, z)$ within the gap between the lens and coverglass, we proceeded to calculate the concentration of the charged probe molecules in the gap. The local

concentration of probe (fluorescent dye) molecules in the gap can be described by the Boltzmann distribution. Assuming a bulk probe concentration c_p, the spatial varying probe concentration can be written as:

$$c_\mathrm{p}(r,z) = c_\mathrm{p} e^{-\frac{q_\mathrm{eff}\phi(r,z)}{k_\mathrm{B}T}} \tag{S7}$$

where q_eff is the effective charge of the probe molecule and $\phi(r,z)$ is the electrical potential distribution in the gap(5-10). The experimentally measured image of the dye emission is radially symmetric yielding a radially dependent intensity $I(r)$ measurement. The intensity measured in the image plane corresponds to a 2-D projection of the 3-D dye distribution in the gap. Integrating Eq. (S7), we obtain an expression for the theoretically expected radial intensity profile given as follows:

$$\begin{aligned} I(r) = \beta \int_0^{H(r)} c_\mathrm{p}(r,z)\, dz = \beta c_\mathrm{p} \int_0^{H(r)} e^{-\frac{q_\mathrm{eff}\phi(r,z)}{k_\mathrm{B}T}} dz \\ = \beta c_\mathrm{p} \int_0^{H(r)} e^{-\frac{q_\mathrm{eff}\psi(r,z)}{e}} dz \end{aligned} \tag{S8}$$

We use a proportionality β to relate the expected intensity $I(r)$ and the dye concentration in order to account for other contributions to the intensity from the experimental setup that we do not directly measure. These contributions include those from the quantum efficiency of the dye and camera sensor, collection efficiency of the objective, the point spread function of the optical system, optical losses within the set-up, etc. For this work, we assume these contributions remain constant over the field of view and that they contribute linear scaling factors outside the integral. Note that because Eq. (S8) is solely a function of ψ and the charge of the probe, q_eff, the solution of the PB equation directly permits us to calculate the expected intensity (within a factor given by the system dependent proportionality constant β). For typical measurement conditions (from 0.01 mM to 1 mM NaCl) β remained fairly constant at $\beta \approx 0.2 \pm 0.01$ for both pH 6 and pH 9. Varying the value of the surface charge density in Eq. (S6), we generate theoretical $I(r)$ curves for a probe molecule of a known charge, and then compare the calculated profiles with the experimental measurements. We are thus able to determine the value of the unknown surface charge density σ and the corresponding electrical potential ψ_s characterizing the surface material of interest.

2.A. Accounting for super-critical angle fluorescence contributions in the collected optical intensity

In order to accurately model the expected intensity profile due to the distribution of fluorescent probe molecules in the gap, we consider the coupling of photons from a dipole close to the two

water-glass interfaces on either side of the solution-filled region. In this sub-section, we describe an optical collection function, $\mathrm{OCF}(z')$, that must be incorporated into Eq. (S8). The primary purpose of the OCF is that it accurately accounts for the total emission collected from the dye which is strongly influenced by the presence of the dielectric discontinuity (water/glass interface) at the upper surface of the gap (Fig. 1*A*, Fig. S4*A*). Eq. (S8) is thus modified to yield

$$I(r) = \beta c_p \int_0^{H(r)} e^{-\frac{q_{\mathrm{eff}}\psi(r,z)}{e}} \times \mathrm{OCF}(z')\, dz \tag{S9}$$

where $z' = H(r) - z$ is the distance of the emitter from the upper glass/water interface. We calculate the OCF of our system as follows. An excited dipole in a medium of refractive index n_1 , which is at a distance $z < \lambda_{\mathrm{em}}$ from an interface with a medium of larger refractive index $n_2 > n_1$ (where λ_{em} is the emission wavelength), will emit Supercritical Angle Fluorescence (SAF), i.e., emission into surface at angles θ above the critical angle of total internal reflection $\theta_{\mathrm{c}} \approx 61°$ at the water-glass interface. This emission occurs in addition to fluorescence at angles below the critical angle (Under-critical Angle Fluorescence, UAF). Here θ is the angle included between a ray emanating from the dipole emitter and the normal vector at the surface substrate (Fig. S4*A-D*) (11, 12). For dipoles located close to the interface, approximately two-thirds of the total fluorescence is emitted into the higher index medium, as the evanescent field of the dipole emitters couples into the denser medium and is transformed into propagating plane waves. These rays propagate at or beyond the critical angle θ_{c} (13). Importantly, the amplitude of the SAF plane waves depends exponentially on the height of the dipole, z', above the layer. Thus, for dipoles more than about a wavelength from the surface there will be virtually no light coupled into directions beyond the critical angle.

2.A.i. Radiating dipole near an interface

Enderlein *et al*. (14) describe the angular emission into the water half-space for a dipole for both vertical and parallel orientations with respect to the plane of the interface as follows:

$$\frac{dS_{1,\perp}}{d\Omega} = \frac{3}{8\pi}\left|\sin\theta + R_p(\theta)\exp(i2n_1k_0z'\cos\theta)\right|^2 |\sin\theta|^2 \tag{S10}$$

$$\frac{dS_{1,\parallel}}{d\Omega} = \frac{3}{8\pi}\left| -\cos\phi\cos\theta + R_p(\theta)\exp(i2n_1k_0z'\cos\theta)\cos\phi\cos\theta\right|^2 + \left|1 + R_s(\theta)\exp(i2n_1k_0z'\cos\theta)\sin\phi\right|^2 \tag{S11}$$

where $\mathrm{d}\Omega = sin(\theta)\mathrm{d}\phi\mathrm{d}\theta$ is the solid angle and the above formulae hold for emission into a homogeneous medium of refractive index n_1. Here, $R_{p,s}(\theta)$ and $T_{p,s}(\theta)$ are the Fresnel coefficients for reflectance and transmittance for p- and s-polarized light.

Similarly, for emission into the glass half-space we have

$$\frac{dS_{2,\perp}}{d\Omega} = \frac{3n}{8\pi}\left|\frac{\cos\theta}{w(\theta)}\right|^2 \exp\big(2Im\big(w(\theta)\big)z'k_0\big) \,|\, \frac{T_p(\theta)n_2}{n_1}\sin\theta|^2 \tag{S12}$$

$$\frac{dS_{2,\parallel}}{d\Omega} = \frac{3n}{8\pi}\left|\frac{\cos\theta}{w(\theta)}\right|^2 \exp\big(2Im\big(w(\theta)\big)z'k_0\big)\,(|\frac{T_p(\theta)}{n_1}w(\theta)\cos\phi\,|^2 + \,|T_s(\theta)\sin\phi\,|^2) \tag{S13}$$

The total emission into either half space can be calculated by integrating over a solid angle:

$$S_i(z') = \int_{-\pi/2}^{\pi/2}\int_0^{2\pi}\frac{\mathrm{d}S_i}{\mathrm{d}\Omega}\sin\theta\,\mathrm{d}\theta\mathrm{d}\phi \tag{S14}$$

where S is the total emitted power into one of the half spaces, with $i\; = 1$ and 2 denoting water and glass respectively.

For a radiating dipole with a random orientation we then have:

$$< S_i(z') > = \frac{1}{3}S_{i,\perp} + \frac{2}{3}S_{i,\parallel} \tag{S15}$$

In the experiment we collect emission up to collection angles given by the numerical aperture (NA) of the objective. We therefore integrate over an interval of the polar angle, $\theta_{\mathrm{NA}} = \sin^{-1}\left(\frac{\mathrm{NA}}{n_i}\right)$, yielding an Optical Collection Function (OCF)

$$\mathrm{OCF}(z') = \int_{-\theta_{\mathrm{NA}}}^{\theta_{\mathrm{NA}}}\int_0^{2\pi}\frac{\mathrm{d}S_i}{\mathrm{d}\Omega}\sin\theta\,\mathrm{d}\theta\mathrm{d}\phi \tag{S16}$$

2.*A.ii*. Estimation of the collected emission in the experimental setup.

We indicated in the previous section that the OCF primarily accounted for influences on the dipole emission arising from the “upper” surface of the gap. We discuss here why distance of the emitter from the lower half space does not strongly influence the total collected photons. In Fig. *S4A,C-D* we see that for dipoles close to an interface, the emission into the glass does increase dramatically with distance. However, this increase in emission arises from light propagating due to the

evanescent field of the emitter, which is directed entirely at angles equal to and greater than critical angle. The emission into the undercritical angle region on the other hand remains unaltered. Therefore, in experiments such as ours where NA<1.33, the collection of emission from the lower dielectric half space has no distance dependence, as indicated by evaluating Eq. (S12) and (S13) in Fig. S4*A*-*D*. In other words, in the regime $\theta \leq \theta_c \approx 61°$, we only collect UAF emission which is independent of the location of the emitter given by z'.
On the other hand, the upper dielectric discontinuity does exert a strong influence on the total collection. This is because the emission into the lower water slab is in fact affected by the presence of the interface and also carries a strong distance dependence, which is true for all collection angles (Fig. S4A).

In our present model, we treat the contribution from the two interfaces (upper glass surface/water and water/coverglass) independently and superpose them in the final result. A more comprehensive model of collected photons in a system containing multiple dielectric interfaces can be developed with the help of finite difference simulations and will be discussed in future work.

2.B. Comparing calculated $I(r)$ profiles with experimentally measured intensity distributions.

Fig. S1*A* illustrates the post-processing of an experimentally acquired snapshot to extract a radially symmetric image of the probe molecule distribution from which we obtain a radially dependent intensity profile $I(r)$. To extract values of surface electrical potential or surface charge density (on the glass and lens surfaces) responsible for the intensity profile, we use a template matching approach that compares the experimental $I(r)$ curve to a set of template intensity curves, that are obtained by solving the NLPB equation for the corresponding salt concentration of the experiment. Each template $I(r)$ curve represents a particular value of constant surface charge σ_i on the confining walls, where σ_i generally takes a value between 0 to 0.1 e/nm^2 (Fig. S5*D*). The radial intensity profile $I_\mathrm{i}(r)$ for a surface charge density value σ_i is calculated using Eq. (S9). The calculated $I_\mathrm{i}(r)$ curves are matched to the measured intensity profile $I(r)$, using a multiplicative scaling factor, β, as a single fit parameter and minimizing the residuals between the calculated and measured intensity curves (Fig. S5*D*). For typical measurement conditions (from 0.01 mM to 1 mM NaCl) β remained fairly constant at $\beta \approx 0.2 \pm 0.01$ for both pH 6 and pH 9. The residuals between the scaled $I_\mathrm{i}(r)$ and measured $I(r)$ are plotted (Fig. S5*E*) in order to determine the value of surface charge which yields the smallest residual. In the case shown in Fig. S5*B* and *D*, a minimum is found for a σ_i value of ~0.015 e/nm^2 for SiO_2 at pH 9 and ~0.1 mM salt. The obtained value of surface charge can then be uniquely related to values of surface electrical potential, ψ_s, as shown in Fig. S5*F*. The only experimental parameters required for the data analysis procedure are the measured conductivity of the solution and the interferometrically determined height profile H.

In future work, the potential distribution calculations with the PB equation could be extended to go beyond the constant potential or constant charge model and include charge regulation, finite ion size, and other contributions to the total interaction e.g., hydration forces.(15-17)

3. Extracting the chemical species properties of the silica surface using measurements of surface potential at pH 4.5, 6 and 9

Silica surfaces acquire electrical charge primarily through the dissociation of silanol (SiOH) groups in contact with an electrolyte, as illustrated by the following reaction:

$$\mathrm{SiOH} \rightleftharpoons \mathrm{SiO^-} + \mathrm{H^+} \tag{S17}$$

Silica surfaces are thought to be characterized by at least three different types of ionizable species *i* with characteristic acid dissociation constants $\mathrm{p}K_i$ whose extent of deprotonation, α_i, depends on the solution pH(18-21). In order to understand the charging process responsible for the surface potentials measured in our experiments (Fig. 2), we generalize the method reported by Behrens and Grier to calculate the electric charge density of silica surfaces in contact with aqueous electrolytes(22). In general, we can write the total charge density at the silica surface as follows:

$$\sigma = e\Gamma \sum_{\mathrm{i}} z_i f_i \alpha_i \tag{S18}$$

where *e* is the elementary charge, Γ is the number of silanol groups per surface area and f_i is the fractional preponderance of the ith species, with $\sum_i f_i = 1$. The ionisation probability α_i in turn can be written as:

$$\alpha_i = \frac{1}{1 + 10^{z_i(\mathrm{pH} - \mathrm{p}K_i)} e^{z_i \psi_s}} \tag{S19}$$

where $z_i = \pm 1$ denotes the sign of the charge of the surface group in the ionised state (15). We note that α_i depends on pH and the dimensionless surface (or local) electrical potential ψ_s, which can significantly influence the dissociation process. This phenomenon is known as charge regulation(15). Eq. (S18) and (S19) describe the dependence of the surface charge on the surface electrical potential when chemical reactions govern the charging process. The surface charge also depends on surface electrical potential via the PB equation, a model that describes the screening of the surface potential by counterions in the electrolyte. An exact solution of the NLPB equation for surface charge density as a function of the surface electrical potential of an isolated planar surface is given by the Grahame equation:

$$\sigma(\psi_s) = \frac{2\varepsilon\varepsilon_0\kappa k_B T}{e}\sinh\left(\frac{\psi_s}{2}\right) \qquad \text{(S20)}$$

Our aim in this section is to describe how to determine values for the surface density of ionizable groups (silanol) Γ, as well as the $\mathrm{p}K_i$ and the fractional preponderance f_i of the ionizable species i using opto-electrostatic measurements of the surface charge density, σ, at various pH values. We simultaneously solve Eq. (S18) and (S20) to obtain an electrical surface charge σ_{model}, which we can compare with the σ obtained from the experiments for a given measurement pH and salt concentration in solution. We vary the model parameters $\Gamma, \mathrm{p}K_i$ and f_i in order to minimize the difference between the measured σ and σ_{model}. The standard deviations of the measurement are included as weights in the optimization process.

We first validate the method outlined above using simulated data as inputs (Fig. S7). In Fig. S7*A*, the pH-dependence of σ is calculated for a fictitious model surface whose properties we assume are described by values $\mathrm{p}K_\mathrm{a} = 3$, 6 and 12, as well as a fixed density of ionizable surface groups, $\Gamma = 0.8$ nm^{-2}, with fractional distributions $f_1 = 0.1$, $f_2 = 0$ and $f_3 = 1 - f_1 - f_2$. The aim of the data analysis validation procedure is to recover these known inputs using knowledge of the corresponding charge density σ values alone. In other words, we used calculated σ values as measured inputs and included uncertainties corresponding to 10% of the value in each case in order to simulate measurement uncertainty. The blue points and corresponding error bars in Fig. S7*A* represent these simulated points for our test surface material.

In our analysis procedure we have 6 independent unknown quantities for a trimodal $\mathrm{p}K_\mathrm{a}$ distribution, which are $\Gamma, \mathrm{p}K_i$ and f_i, where $i = 1$–3. In order to solve for the values of the 6 unknowns, we sweep through a range of values for these 6 parameters and calculate the surface charge based on the NLPB charge regulation model. We then estimate the total absolute residual between the measured surface charge value and the calculated result. These residuals are weighted by the standard deviation of the measurement, $\Delta\sigma$, as described in Fig. S7. A global minimum in the residuals is found between the measured surface charge values and the corresponding calculated value from the NLPB charge regulation model. We show in Fig. S7*B-F* that this approach successfully recovers the ground truth surface property parameter values in a validation procedure based on simulated measurement values.

We then apply the same data analysis procedure to surface charge density values inferred from experimental measurements. We model our experimental silica surfaces with a trimodal $\mathrm{p}K_\mathrm{a}$ distribution given by 3 types of ionizable groups whose $\mathrm{p}K_i$ values are approximately 3, 6 and 9.(18, 19, 21) Furthermore, the total group density and number of active dissociating sites for each

species i depend on experimental condition, and sample treatment history. In this work we reported estimates of the total silanol group density Γ, the fractions f_i, and acid dissociation constants $\mathrm{p}K_\mathrm{i}$ of each species assuming that the surfaces we examine do not change their properties depending on treatment history. We use data obtained for surface charge density under 7 different experimental conditions from pH 4.5 to pH 9 and salt concentrations of 0.01, 0.1, and 1 mM as presented in Fig. S8. We used the residual minimization procedure described above to extract values of the 6 unknown parameters of interest. We obtained silanol group density of Γ = 0.13 $\pm$ 0.01 nm^{-2}, as shown in Fig. S8*B* and *C*. Furthermore, within a trimodal $\mathrm{p}K_\mathrm{a}$ description we found that the data pointed to the values $\mathrm{p}K_1 \leq 3$, $\mathrm{p}K_2 = 7.2 \pm 0.8$ and $\mathrm{p}K_3 = 9.65 \pm 0.10$, where the third ($i = 3$) least acidic species appeared to be the most abundant with $f_3 \approx 0.90$. We further found that $f_1 = 0.06 \pm 0.01$ and $f_2 = 0.04 \pm 0.01$ which implies that relatively small fractions of groups with $\mathrm{p}K_1$ and $\mathrm{p}K_2$ appear to be responsible for a non-negligible surface charge at lower pH, i.e. at conditions under which the groups at $\mathrm{p}K_3$ would be expected to be completely uncharged.

3.*A*. Estimating the uncertainty in the determination of $\mathbf{\Gamma}, \mathbf{f_i}$ and $\mathbf{pK_i}$.

The uncertainty determination on each of the inferred parameters can be illustrated with the example shown in Fig. S8*B-D*. In each case, one of the parameters in the 3D parameter space of Γ, f_1 and $\mathrm{p}K_3$ was fixed to the value that minimized the overall deviations between the model and the measured electrical surface charge. The resulting 2D surface plots are presented in Fig. S8*B*, *C* and *D*, respectively, where a minimum for the remaining two parameters was found. The reported uncertainties on Γ, f_i and pK_i were defined as the deviation of each parameter from the mean value required to increase the total residual by 10% from the global minimum residual value.

4. Characterization of effective charge for fluorescent probe molecules using surfaces with a known surface potential $\boldsymbol{\psi_\mathrm{s}}$

Having characterized the surface potential ψ_s of the silica surfaces in the lens-coverglass gap as described in the main text and in *SI Appendix,* Sections 1-2, we used the system to characterize the effective charge q_eff of fluorescent molecules such as organic dyes and single-stranded (ss) DNA (Fig. 2*D*, Fig. S9). Solving for q_eff in Eq. (S9) using the known silica surface potential ψ_s, we obtained measurements of estimated q_eff values for each probe molecule as shown in Fig. 2*D*. In the case of the organic dyes, we note that $q_\mathrm{eff} \approx q_\mathrm{str}$ as is expected for low object charge densities.(5, 7) Furthermore, the estimated q_eff values were in good agreement with the charge values reported by the manufacturer.(23) In the case of the ssDNA, a molecule with a high linear charge density, we found that q_eff was smaller than q_str, which is consistent with the predictions from charge renormalization and counterion condensation theories (5, 7, 24-26). Fig. S9*A* displays

fluorescence images of each species of probe molecules in the lens-coverglass gap, with the corresponding $I(r)$ curves for each image presented in Fig. S9*B*.

5. Measurement uncertainties in the determination of surface electrical potentials

The uncertainties in our reported surface potential values, shown as separate colored and black error bars for each measurement in Fig. 2*C*, correspond to the observed variability in our experiments and in the data analysis, respectively. This section explains these sources of error and describes how the data were processed to yield mean values and their spread in Figs. 2*C* and 3*H*. The experimental error in the reported values of surface potential results both from: (a) sample-to-sample variability, i.e., variability from independent experimental repeats measured on independent samples consisting of freshly prepared lens-coverglass substrates and freshly mixed electrolyte solutions, and (b) from possible variability due to conductivity and pH drift of the solution in the gap with time during each experiment. Three independent measurements were conducted for each experimental condition (pH and salt variation), and during each measurement, at least two different areas, or fields of view (FOV), of the coverglass substrate were imaged. Because the FOVs were imaged sequentially by placing the lens at a different region on the glass surface there was a time delay between measurements that could cause the conductivity and the pH of the solution in the gap to drift, affecting the value of the estimated surface potential. The experimental variation in measured values arising from the order in a measurement sequence (categorical on the x axis) and FOV (box plots) for the data acquired for silica at pH≈6 in an electrolyte containing 0.1 mM NaCl are shown in Fig. S6.

An additional source of error arises from the data analysis procedure and involves a variation in the estimated surface potential arising from the range of gap heights or radii chosen to match the $I(r)$ profiles as described in *SI Appendix,* Section 1. Since the lens-coverglass system provides a variable-height gap, we can select a range of heights, or equivalently, a radial range to match the intensity profiles $I(r)$ extracted in each experiment. As stated in the main text, this height range was chosen based on a physical parameter, the Debye length (κ^{-1}), with a lower bound H_l = $1\kappa^{-1}$ and an upper bound H_u ranging from $2\kappa^{-1}$ to $3\kappa^{-1}$ for 0.01 mM NaCl (pure water) solutions, and H_l = $3\kappa^{-1}$ and H_u ranging from $8\kappa^{-1}$ to $14\kappa^{-1}$ for 0.1 and 1 mM NaCl solutions. These H_u bounds correspond to an approximate height of H~ 300 nm and radius $r\sim$ 100 µm for 0.01 mM NaCl (pure water) solutions, H~ 400 nm and radius $r\sim$ 112 µm for 0.1 mM NaCl solutions in the gap. Beyond this gap height and corresponding radial location in the image, the increasing noise from the corners of the image FOV makes the $I(r)$ unsuitable for extracting surface potentials. Furthermore, heights beyond the upper bound e.g., H_u = 2-$3\kappa^{-1}$ for $c\ \leq$ 0.01 mM and H_u = $14\kappa^{-1}$ for $c\ \leq$ 0.1 are in general not expected to yield reliable data for the measurement of surface potential, as the

electrostatic contribution to the expected optical intensity would be negligible. At heights smaller than the lower bound H_l < $1\kappa^{-1}$ for c $\leq$ 0.01 mM NaCl solutions, and H_l < $3\kappa^{-1}$ for c $\leq$ 0.1 mM NaCl solutions, the uncertainties in the interferometric height measurement (~5 nm) are expected to generate a large uncertainty in the assumed gap height H which would strongly impact the inferred surface charge density and potential values. At least five different height ranges were used for each dataset during the template matching with the calculated $I(r)$ curve, which resulted in some variation in surface potential values for each measured FOV. This spread as a function of varying upper height bound H_u is shown in Fig. S6 as individual, spatially offset data points overlaid on the boxplots for each sampled FOV in each independent measurement.

6. Constructing surface electrical potential and charge density images using the scanning probe system

We used the following procedure in order to convert measured fluorescent intensity distributions obtained using the scanning probe system into estimated spatial distributions of surface charge density and potential on the substrate (Figs. 5*E*-*F*). We initially assume a value of surface charge density, σ, for the "square hole" (silica) regions of the substrate as well as the upper platform or probe surface. Prior knowledge of this value is not necessary as described later, but for this analysis we use an initial value $\sigma = -0.0075\ \mathrm{e/nm^2}$ known from previous measurements in the lens-based system for glass surfaces (Fig. 2). First, we construct simulated 2D images of intensity for a 2D surface carrying a heterogeneous charge density. Images are calculated for specific gap heights, H= 50 nm and 500 nm, which are known independently from interferometric measurement. We then apply an additive background signal level as well noise to each pixel in order to simulate the experimental situation closely. Next, we apply a Fourier filtering step to remove the low frequency components of the image, which is the same as the procedure used to process the experimentally measured images. Varying the charge density in the "test" regions (TiO_2 lines) from 0.001 e/nm^2 to 0.1 e/nm^2 in the simulated images gives us a range of expected interest contrast levels for these regions with respect to the reference SiO_2 surface. We then treat these contrast levels as a library or look-up-table of optical contrast values which can be directly related to surface electrical properties. Next we examine the experimentally measured images at the pixel-by-pixel level and convert these local contrast values to surface electrical potential and corresponding charge densities suggested by our look-up-table of contrasts. In a subsequent step, we note that varying the charge density of the more strongly charged silica regions, which contributes to the background intensity, permits us to simultaneously fine-tune the initial "guesstimate" values for the charge density in these regions. This is because the ratio of measured or calculated optical contrasts at two different heights depends on both the properties of the silica and TiO_2 regions, as the observed intensity values are governed by Eq. (S9) and the Fourier filtering step only permits us to measure

the intensity increment relative to the SiO_2 background. Note that this data analysis approach, exploiting the optical contrast between two different regions of the substrate, is specific to this type of experiment. In general, measurements of the intensity distribution at multiple different gap heights will support extraction of pixel-wise charge density values without recourse to referencing to a particular region of the substrate.

7. Simulations of minimum feature size determination using opto-electrostatic imaging

In order to explore the minimum feature size that could be discerned by means of our opto-electrostatic imaging technique, we performed simulations of the expected signal-to-noise ratio (SNR). Here we define SNR as the ratio between difference in fluorescence signal between two regions, e.g., the TiO_2 features and the SiO_2 substrate, and the noise on the SiO_2 region. The simulations were performed for different values of solution pH, probe charges, and surface potential differences between materials, varying the dimensions of the electrostatic feature of interest on the substrate surface (Fig. S10). Specifically, we address two key aspects regarding the spatial resolution of our method (Fig. S10*A*): the use of highly charged probes and its effect on SNR (Fig. S10*B*-*C*), as well as the sensitivity of the technique to small differences in surface potential between the feature of interest and the background (Fig. S11*C*-*E*). All presented results were determined computationally with a finite element method (FEM) simulation using COMSOL. Intensity noise based on the measured noise from the experimental results was added to the simulated images.

In Fig. S10*A*, we analyze the image contrast created by an uncharged circular feature on a silica surface (under conditions where silica is known to carry a high negative surface charge). The diameter of the feature is varied from 20 – 100 nm. The electrical potential distribution in the structure is obtained using an axisymmetric simulation in COMSOL using a salt concentration of 0.1 mM ($\kappa^{-1} \sim 30$ nm) and a gap height of $H = 110$ nm. A simulated experimental image was generated by convolving an intensity distribution given by Eq. (S8) with a simulated point spread function (estimated using the microscope objective NA, the emission wavelength of the fluorophore, and the pixel size of the camera) to obtain the images shown in Fig. S10*A*. A discernible contrast in the image can be observed for features approximately $s = 2\kappa^{-1}$ in radius, although features of even $s = 1\kappa^{-1}$ radius could potentially be visible in a measurement under the investigated conditions. The ability to detect smaller features can be improved by reducing the Debye length (using a higher salt concentration) and by scaling the height proportionally, such that $\kappa H \approx 3.7$.

Furthermore, simulations of the experiments reported in Fig. 4 and Fig. 5 confirm the observed results and the expectation that molecular probes carrying higher values of net charge ($|q_{\mathrm{str}}| > 1e$) may be used to enhance optical contrast due to electrostatics (Fig. S10*B*-*C*). To further explore this property, we evaluate the SNR of a simulated 500 nm TiO_2 feature on glass at 0.1 mM salt

concentration ($\kappa^{-1} \approx 30$ nm) and vary the gap height H of the system. We find a maximum SNR for all probes occurs at a height $H \approx 110$ nm, with a significant increase in contrast for highly charged probes. We note a 4-fold increase of the SNR going from a probe with a single charge -1e to -8e charges. If we require the minimum resolvable feature size to have an SNR of 2, then Fig. S10*C* shows that a dimension of at least $\approx 2\kappa^{-1}$ is required. Interestingly a saturation of the SNR is achieved with a feature radius r > 200 nm (independent of the probe charge), assuming a fixed gap height of 110 nm. We attribute this result to the feature size exceeding both the Debye length and the width of the point spread function.

These simulations can be extended to estimate the optical contrast arising from heterogenous patterns of arbitrary thin film materials, using the surface potential values estimated from homogeneous thin film measurements for various pH conditions (Fig. 3). Here contrast is defined as the difference between the intensity of a less charged surface (appearing brighter due to greater local dye concentration) and a highly charged surrounding surface (appearing darker due to a low dye concentration). Our experimental results showed that the optical contrast due to electrostatics between TiO_2 (grid) and SiO_2 (substrate) is high at pH≈6 and low at pH≈9 (Fig. 4 and Fig. 5) using a dye of charge $q_{\mathrm{str}} = -3e$ (Atto 542c). We simulated the expected SNR for a TiO_2 grid on a SiO_2 substrate (Fig. S11*A-B*) using the surface potential values measured in homogenous thin film substrates (Fig. 3). The simulation (Fig. S11*B*) showed that a significant contrast (SNR ≈ 4) between TiO_2 and SiO_2 is observed at pH 6 and reduced contrast is observed at pH 9 (SNR ≈ 2) using a dye of -3e charge (Atto 542c), which agrees qualitatively with our experimental results (Fig. 4*A-C* and Fig 5*E-H*).

Finally, we examine the measurement of small electrical surface potentials in Fig. S11*C-E*. The SNR analysis outlined above was repeated for features whose surfaces were at a small potential difference of $\Delta\psi_{\mathrm{s}} = \pm 0.5$ with respect to the surrounding substrate surface. We observed the maximum achievable SNR in such systems does not increase even with the use of highly charged probe molecules. However, increasing probe charge causes the gap height at which the peak in the SNR occurs to shift to larger values. This means that although the ability to optically detect small differences in surface potentials is not significantly improved, highly charged probes would permit the detection of a similar level of image contrast at larger gap heights or effectively larger κH. We further note that the contrast (SNR) is larger for negative values of $\Delta\psi_{\mathrm{s}}$.

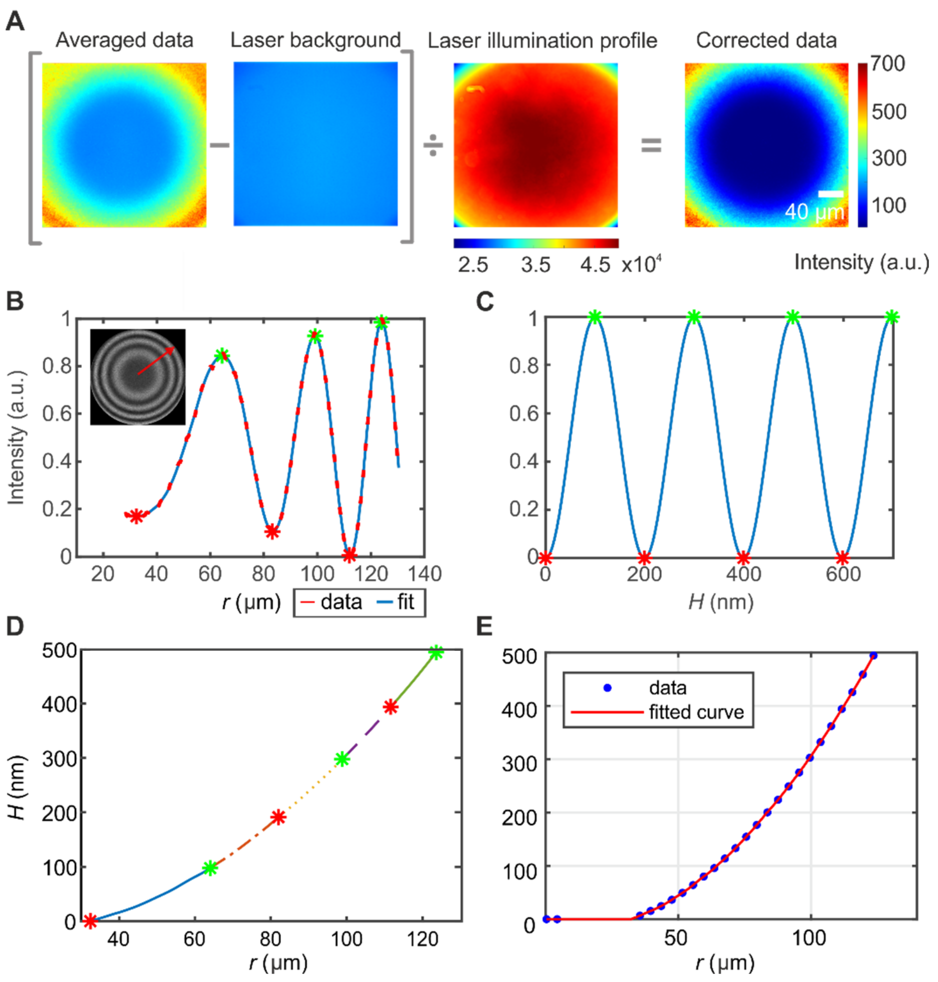

Fig. S1. Image processing procedures for the correction of non-uniform illumination profile in fluorescence imaging, and for interferometric determination of the slit height. (*A*) An image of the laser excitation background, acquired with pure water (no dye) on the coverslip with no lens on top, is subtracted from a raw, widefield fluorescence image of dye solution in the lens gap. The resulting image is divided by an image of the illumination profile (obtained by imaging a drop of dye solution on the coverslip without the lens on top) to obtain a background and illumination-corrected image. (*B*) Radially averaged cross-section through an interferometry image (shown as an inset), with a polynomial fit of intensity shown as a solid blue line on the data (red line). (*C*) Calculation using the Fresnel equations (Eq. S1) of the spatial profile of imaged intensity including minima and maxima as a function of height H inside the gap. (*D*) Piecewise interpolation of gap height by matching the measured (*B*) and calculated (*C*) intensity traces over ranges bounded by each successive pair of minima (red stars) and maxima (green stars). (*E*) Measured gap height H as a function of radial distance from the center of the lens.

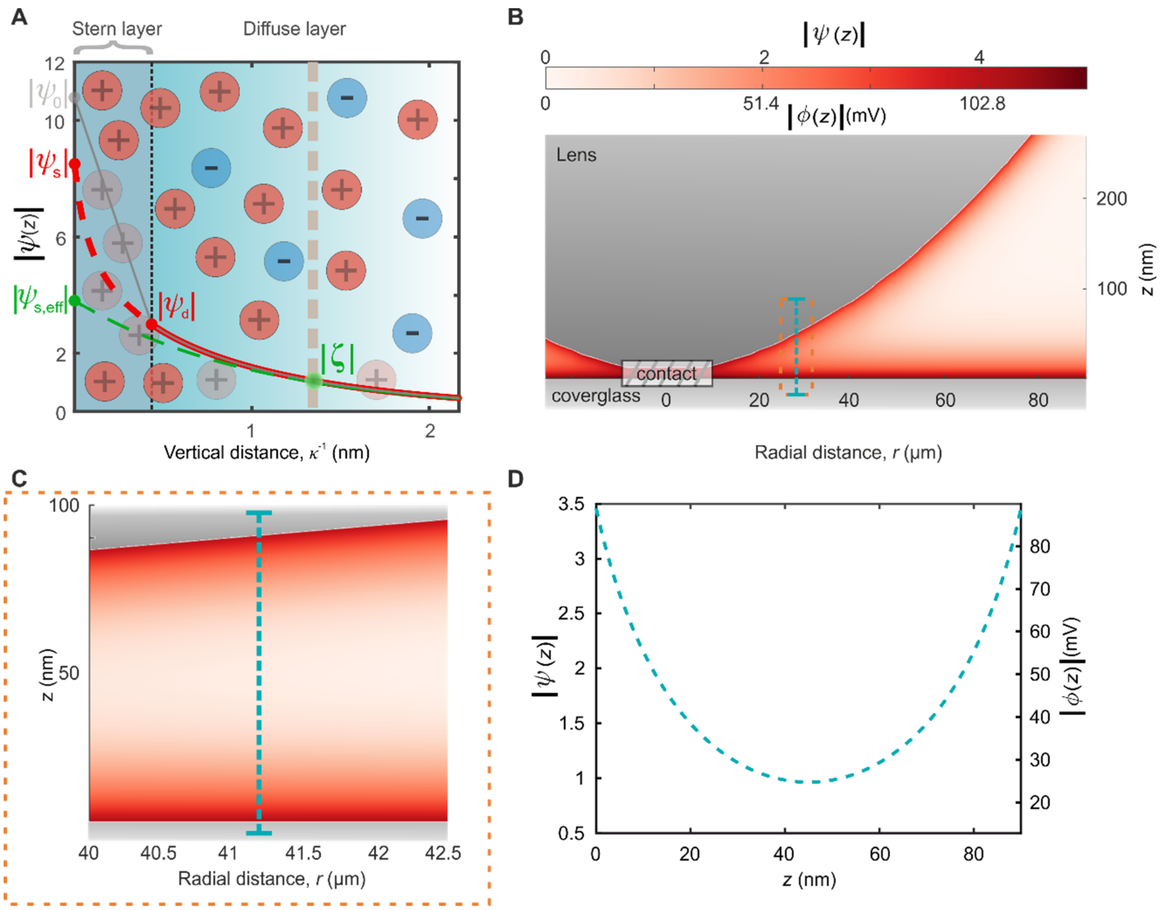

Fig. S2. Electrical potential distribution in a variable height gap. (*A*) Schematic representation of an electrolyte at a negatively charged surface. The cations in the solution screen the negatively charged wall creating a Stern Layer at the interface and a subsequent diffuse counterion layer. The analysis presented in this work is based on electrical potentials calculated using the non-linear Poisson Boltzmann equation (NLPB, Eq. (S5)) (red lines), which accurately describes the diffuse layer. ψ_s is the dimensionless electrical potential in a model of the surface excluding the Stern layer (27, 28). Note that in a model which includes the Stern layer, ψ_0 is the Stern Layer potential and ψ_d is the potential at the boundary to the diffuse layer. As shown in Eq. 1, the electrical potential at large distances from the wall can be approximated by the exponential decay of an effective surface electrical potential, $\psi_{s,eff}$, (green line) which in turn can be similar in value to the Zeta potential, ζ (29). (*B*) Lens-coverglass system creating a variable height gap where both top and bottom silica surfaces acquire a charge in solution. Electrical potential distribution in the gap calculated based on the NLPB equation for an aqueous solution containing c = 0.01 mM monovalent salt (red color scale; red lines in (*A*)). Note that we do not determine the potential distribution in the contact region where the uncertainty of the gap height is too large. (*C*) Magnified view of the region depicted in (*B*). Due to the small curvature of the lens, we can approximate the system as a parallel plate gap to calculate the axial potential distribution in z at each radial position,

r. (*D*) Axial potential profile in the gap calculated for a cross section denoted by the blue dashed line in (*B*-*C*).

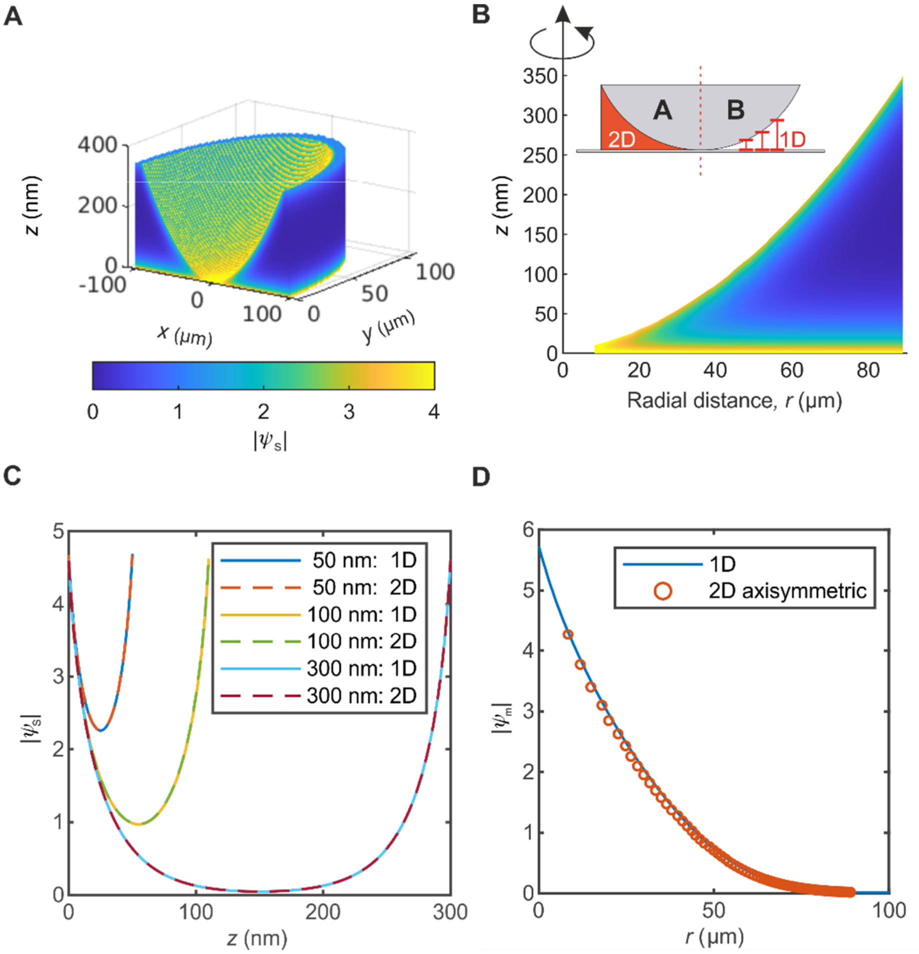

Fig. S3. Axisymmetric vs 1D simulation of the lens-based system. (*A*) Result of an axisymmetric simulation of the NLPB equation showing the potential distribution in the region of small radii ($r < 100$ μm) for a silica lens in contact with a silica substrate in an electrolyte containing $c = 0.1$ mM monovalent salt, where the surfaces have a uniform charge density of $\sigma = 0.035\ e/\text{nm}^2$. (*B*) Potential distribution in the lens-coverslip gap as constructed from series of solutions to the NLPB equation in a 1D system with variable gap height H. (*C*) Potential distributions at 3 nominal heights, $H = 50$ nm, 100 nm and 300 nm, where the solid lines represent the 1D simulations and dashed lines represent the 2D axisymmetric simulation. Agreement between the two is within numerical precision of the solution (~0.5% deviation for selected 2D mesh density). (*D*) Comparison of the midplane potential value, ψ_m, at the center of the gap ($z = H/2$), in both models. The average deviation between the two results is ~ 4 %.

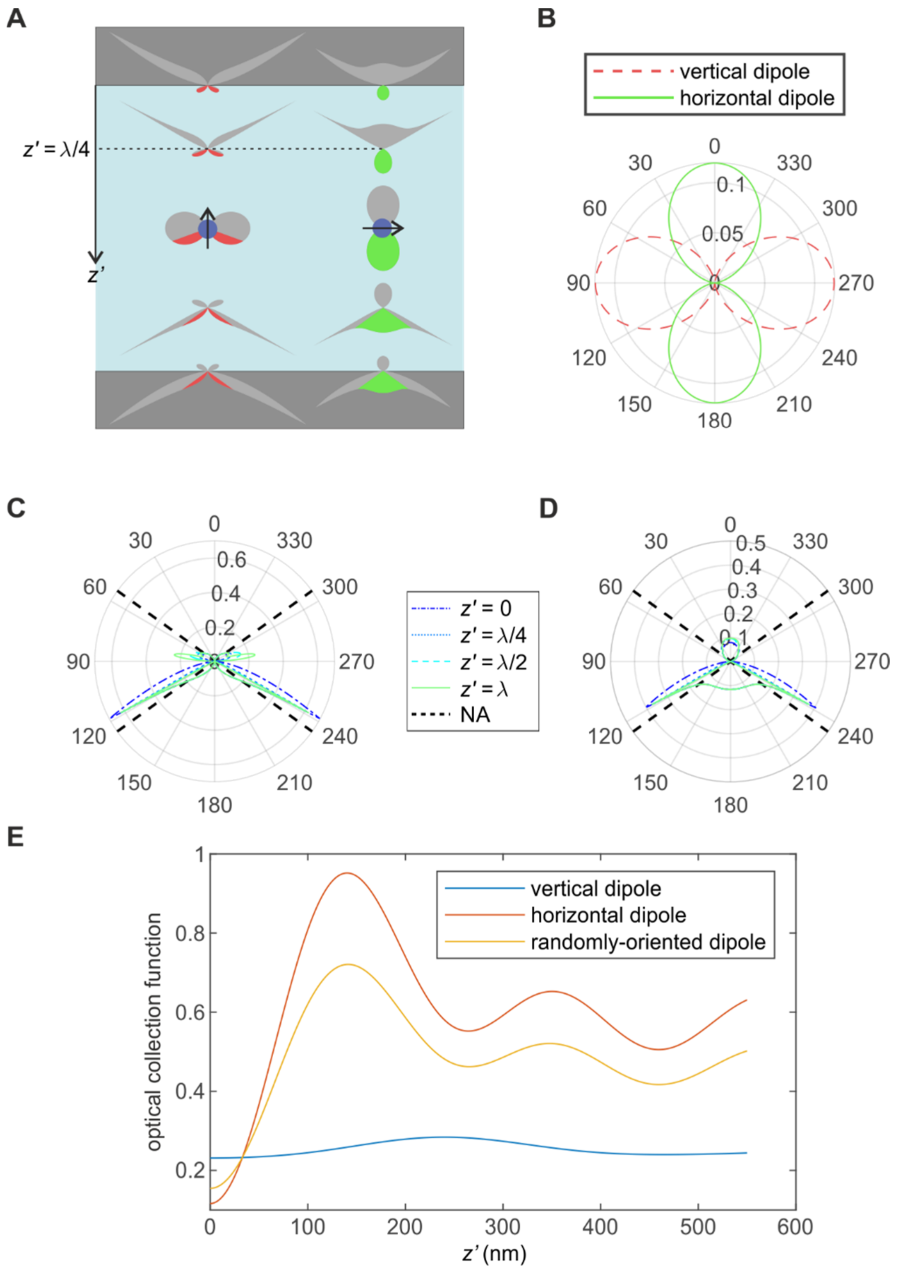

Fig. S4. Angular distribution of emission for a radiating dipole near a water/glass interface. (*A*) Angular emission patterns for a fluorescent emitter, modelled as a radiating dipole, at various distances z' from the lens surface. The emitted light is collected from the bottom (coverglass side) with an objective of NA = 1.25. The angular properties of the collected emission for vertically and horizontally oriented dipoles are colored red and green respectively. (*B*) Angular distribution plot of the emitted power for vertical and horizontal dipoles several wavelengths away from the interface. (*C*) Angular distribution plot of a vertical dipole's emission near a water/glass interface. (*D*) Angular

distribution plot of a horizontal dipole's emission near a water/glass interface. (*E*) Total collected emission from a radiating dipole as a function of distance from the top surface, z'.

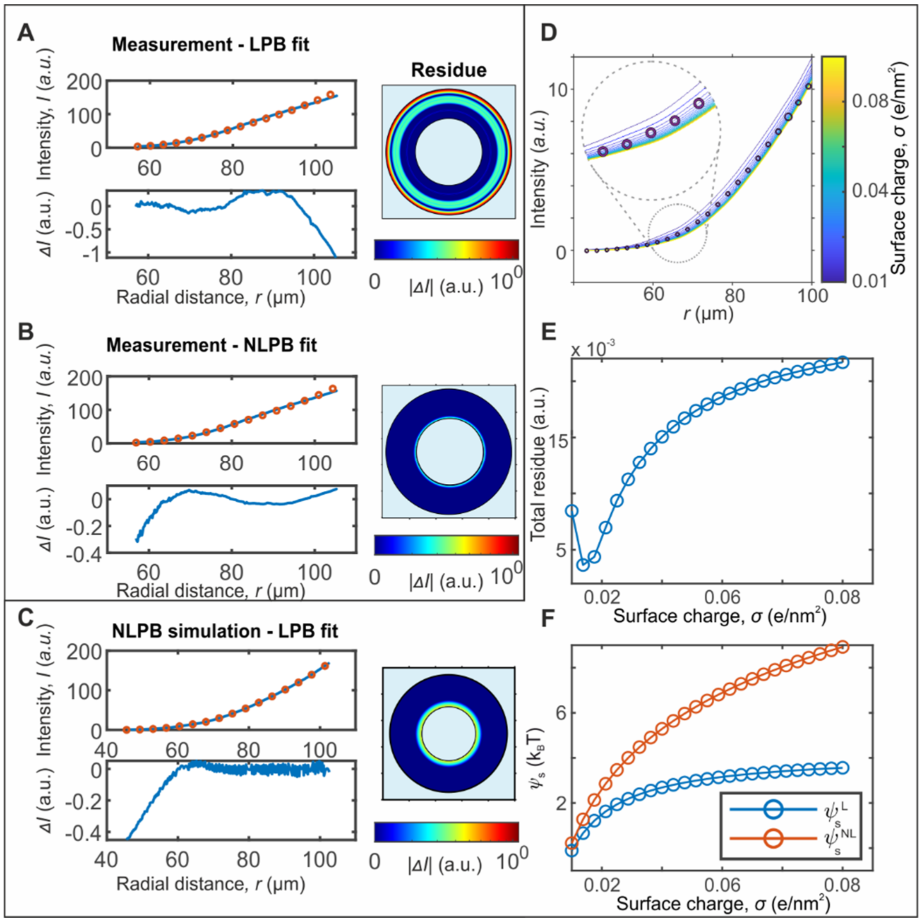

Fig. S5. Comparing measured radial fluorescence intensity distributions with the Linear (LPB) and Nonlinear Poisson-Boltzmann (NLPB) model to estimate surface charge density and surface potential of the substrate. (*A*) Fit of a radial intensity curve measured at pH≈9 and 0.1 mM NaCl with the LPB model. The magnitude of the difference between experimentally recorded and the fitted intensity distribution is shown on the right. (*B*) Fit of a radial intensity curve measured at pH≈9 and 0.1 mM NaCl with the NLPB model. (*C*) Fit of a simulated measurement (with the NLPB model) with the LPB model. Because the “LPB fit” of intensity data simulated using the NLPB model does not yield perfect agreement we use the NLPB model to fit all our measured data. Note that Panels A and B clearly show that the experimentally measured intensity data fare better when treated with the NLPB model (smaller fit residuals). (*D*) Intensity profiles (lines) calculated using the interferometrically-determined geometry of the gap for increasing surface charges from 0.01 to 0.1 e/nm^2. A measured intensity trace (circular symbols is overlaid on these calculated profiles, with the radius of the circle representing the uncertainty in the measurement. (*E*) Plot of the residuals, determined by a weighted least-squares method, between each calculated constant charge intensity curve and the measured data from (*D*). We obtain a minimum at $\sigma \approx -$ 0.015 e/nm^2 for the experimental data (circular symbols) in (*D*), which corresponds to an electrical

surface potential of $\psi_s \approx -3$ at 0.1 mM NaCl. (*E*) Surface potential value as a function of surface charge from the NLPB equation.

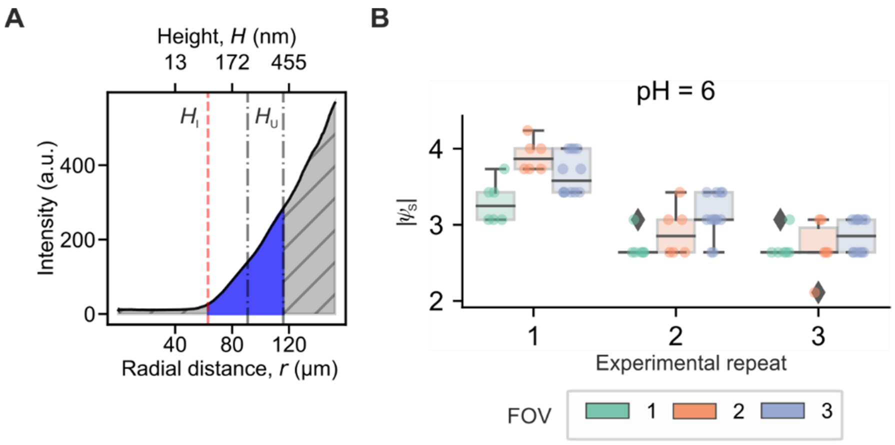

Fig. S6. Measurement uncertainty in surface electrical potential measurements. (*A*) Representative $I(r)$ curve plotted for the pH≈9, 0.1 mM NaCl data from Fig. 2*A*, with vertical boundaries showing the height, and equivalent radial range (blue shaded area) used for template matching with the NLPB model. The red dashed line shows the lower height bound H_{l} which is equivalent to ~$3\kappa^{-1}$ for c ~ 0.1 mM NaCl, and the black dot-dashed lines denote upper bounds H_{u}, between $8\kappa^{-1}$ to $14\kappa^{-1}$. (*B*) Summary statistics of the estimated surface potential values for the pH6 0.1 mM NaCl data in Fig. 2*A* and (*C*) showing the experimental variation as a function of repeat (categorical on the x-axis) and FOV (box plots in different colors). Individual data symbols represent variations in the inferred electrical potential value arising from variations in the value of H_{u} in the template matching procedure for a given measured image.

A

Charging model (input)		Simulated data			
Parameters	Values	pH	c (mM)	σ (e/nm^2)	ψ_s
Γ (nm^{-2})	0.8	6	0.01	-0.038	-7.0
pK_1	3	6	0.1	-0.062	-5.7
f_1	0.1	6	1	-0.076	-3.9
pK_2	6	9	0.01	-0.080	-8.5
f_2	0	9	0.1	-0.080	-6.2
pK_3	12	9	1	-0.080	-3.9
f_3	0.9	4.5	0.1	-0.028	-4.1

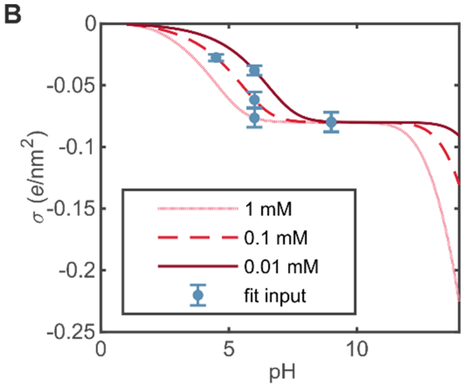

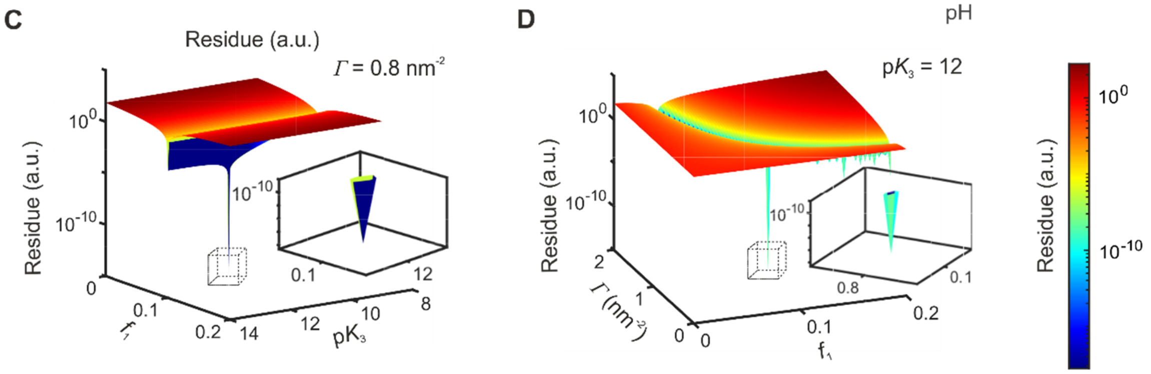

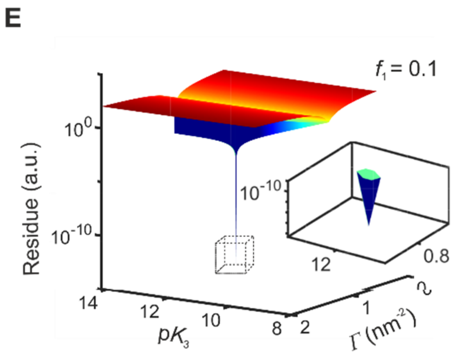

F

Model fit (output)	
Parameters	Values
Γ(nm^{-2})	0.8
pK_1	3
f_1	0.1
pK_2	6
f_2	0
pK_3	12
f_3	0.9

Fig. S7. Validation of the analysis approach used to characterize the surface chemical species properties (Γ, pK_i, f_i) of an arbitrary material using simulated σ data. (*A*) Tabulated summary of the simulated data displayed in (*B*). The data points were generated by solving Eqs. (S18) and (S20) simultaneously for a fixed silanol group density Γ = 0.8, with fractional preponderances f_1 = 0.1, f_2 = 0 and f_3 = 0.9 at acid dissociation constants pK_1 = 3, pK_2 = 6 and pK_3 = 12, which were arbitrarily chosen. For a given condition, specified by the pH and salt concentration c, values for the electrical surface charge σ and electrical surface potential ψ_s calculated from the model were used as simulated measurement points (blue circles in (*B*)). (*B*) Plot of the surface charge density, calculated as a function of pH for the fictitious material with arbitrarily chosen surface property parameters tabulated in (*A*), at salt concentrations $c \approx$ 0.01, 0.1, and 1 mM. The blue circles denote arbitrarily selected calculated values, which correspond to particular values of the underlying set of 6 surface property parameters shown in (*A*). These points

and their corresponding error bars are treated as "simulated measurements" and are used as inputs in the residual minimization data analysis procedure outlined in *SI Appendix,* Section 3. Results of the optimization procedure for 3 out of 6 parameters are depicted in (*C*-*E*). (*C*) Surface plot of the residuals between model and simulated measurement fixed at the optimal value of $\Gamma = 0.8$ nm^{-2} and variable values of both $\mathrm{p}K_3$ and f_1. (*D*) Surface plot of the residuals between model and simulated measurement data fixed at the optimal value of $\mathrm{p}K_3 = 12$ and variable values of both Γ and f_1. (*E*) Surface plot of the residuals between model and simulated measurement data fixed at the optimal value of $f_1 = 0.1$ and variable values of both $\mathrm{p}K_3$ and Γ. The color scale shows the magnitude of the fit residuals at each point in the 2D parameter space, defined by a weighted least-squares method. Specifically, we define the residuals, R, by the sum of the squared differences between the electrical surface charge of the simulated measurements, σ_i, and the model, σ_m, with the weight determined by the corresponding square of the standard deviation of the measurements, $\Delta\sigma_i$, which we formulate with the following equation $R = \sum_{i=1}^{7}\left(\sigma_i - \sigma_{m,i}\right)^2 \cdot \frac{1}{(\Delta\sigma_i)^2}$. The color bar scale is the same for all surface plots (*C*-*E*). (*F*) The inputs subject to the analysis procedure outlined in *SI Appendix,* Section 3 determined the output values for Γ, f_i and $\mathrm{p}K_i$ and are tabulated. The values are found to agree well with the known inputs from (*A*).

A

Experimental data (input)			
pH	*c (mM)*	*σ* (*e*/nm²)	ψ_s
6	0.008	-0.009±0.004	-4.2±0.5
6	0.125	-0.019±0.005	-3.1±0.4
6	1	-0.007±0.006	-0.6±0.4
9	0.125	-0.022±0.014	-3.3±1.0
9	0.175	-0.017±0.009	-2.9±0.8
9	1	-0.025±0.013	-1.8±0.4
4.5	0.158	-0.008±0.004	-1.5±0.3

B

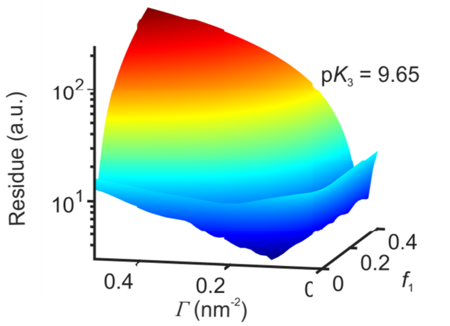

C **D**

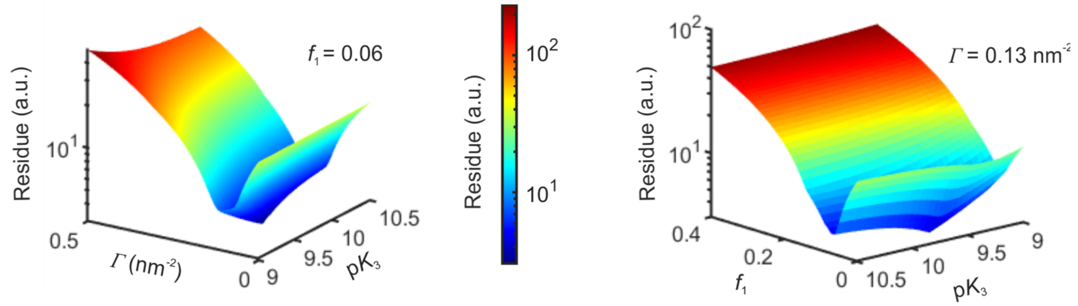

E

Model fit (output)	
σ (*e*/nm²)	Values
Γ(nm⁻²)	0.13±0.01
pK_1	≤3
f_1	0.06±0.01
pK_2	7.2±0.8
f_2	0.04±0.01
pK_3	9.65±0.10
f_3	0.90±0.02

F

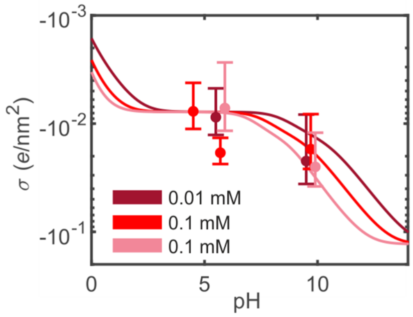

G

Condition		Model predictions	
pH	*c* (mM)	*σ* (*e*/nm²)	ψ_s
6	0.008	-0.008	-4.1
6	0.125	-0.008	-1.9
6	1	-0.008	-0.7
9	0.125	-0.017	-3.0
9	0.175	-0.018	-2.8
9	1	-0.024	-1.9
4	0.158	-0.008	-1.6

Fig. S8. Determining the surface chemical species properties for silica ($\Gamma, \mathrm{p}K_i, f_i$) using measured surface charge density values, σ. (*A*) Tabulated summary of the measured values of σ and ψ_s presented in Fig. 2*C* and in the main text with one additional measurement at pH 4.5. These values with their corresponding error were used as inputs in the residual minimization data analysis procedure outlined in *SI Appendix,* Section 3 and Fig. S7. Results of the minimization for 3 out of 6 parameters are depicted in (*B*-*D*). (*B*) Surface plots of the residuals between model and measured data for fixed $\mathrm{p}K_3 = 9.65$ and variable values of both Γ and f_1. (*C*) Surface plots of the residuals between model and measured data for fixed $f_1 = 0.06$ and variable values of both $\mathrm{p}K_3$ and Γ. (*D*) Surface plots of the residuals between model and measured data for fixed $\Gamma = 0.13$ nm^{-2} and variable values of both $\mathrm{p}K_3$ and f_1. The color scale shows the magnitude of the fit residuals at each point in the 2D parameter space, defined by a weighted least-squares method. Specifically, we define the residuals, R, by the sum of the squared differences between the electrical surface

charge of the measurements, σ_i, and the model, σ_m, with the weight determined by the corresponding square of the standard deviation of the measurements, $\Delta\sigma_i$, which we formulate with the following equation $R = \sum_{i=1}^{7}\left(\sigma_i - \sigma_{m,i}\right)^2 \cdot \frac{1}{(\Delta\sigma_i)^2}$. The color bar scale is the same for all surface plots (*B-D*). (*E*) Tabulated summary of the final result for the values of the underlying set of 6 surface property parameters following the residual minimization data analysis procedure is outlined in *SI Appendix,* Section 3 and Fig. S7. The reported uncertainties on Γ, f_i and $\mathrm{p}K_i$, where $i = 1 - 3$, were defined as the deviation of the parameter value from the minimum at which the residuals increase by 10%. (*F*) Surface charge density σ values plotted as a function of pH. The points with error bars correspond to the measured values of σ presented in (*A*). The solid lines were generated by solving Eq. (S18) and (S20) simultaneously for the minimized Γ, $\mathrm{p}K_i$, and f_i values for silica, where $i = 1 - 3$, and at salt concentrations $c \approx$ 0.01, 0.1, and 1 mM. The minimized surface parameters are tabulated in (*E*). (*G*) Calculated values for σ and ψ_S based on the underlying surface parameters summarized in (*E*) for the measurement conditions in the variable height gap, specified by the pH and salt concentration c (*A*). Note that these values agree well with the measurements used as inputs shown in (*A*). The solid lines in Fig. 2*C* are trends calculated using values of surface properties parameters listed in (*E*).

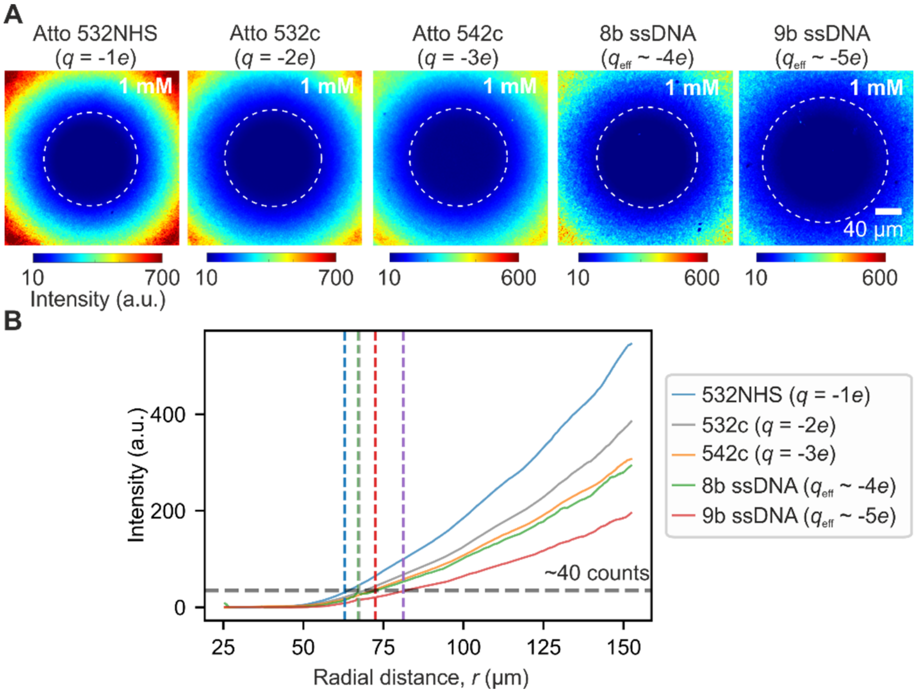

Fig. S9. Images and $I(r)$ curves used for effective charge characterization of fluorescent probes in the lens-coverglass gap using a substrate with known ψ_s. (*A*) Intensity distributions for solutions of fluorescent dye or ssDNA probe molecules in 1 mM NaCl solution at pH 9, with a dashed white contour line at 40 intensity counts for comparison across measurements. (*B*) Intensity traces versus radial distance for each probe shown in (*A*), with a vertical dashed line depicting the radius at which the intensity corresponds to ~40 counts (horizontal dashed line) in each case.

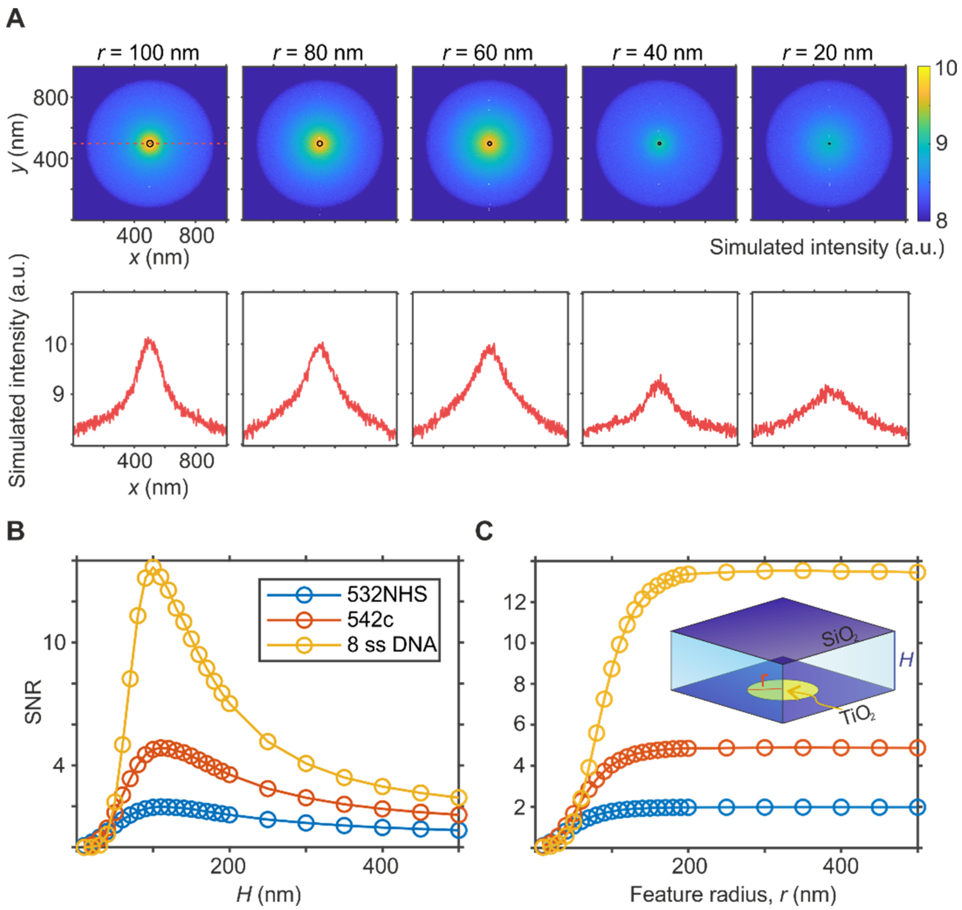

Fig. S10. Simulation-based studies of the minimum feature size and the minimum potential difference that can be visualized on a heterogeneous surface. (*A*) Images calculated using a 2D axisymmetric finite element simulation of a circular feature on a substrate (as depicted in the inset of (*C*)) convolved with a simulated Gaussian point spread function with FWHM ≈ 500 nm at 0.1 mM NaCl and a gap height H = 110 nm. Circular feature sizes varying from 100 to 20 nm in diameter (left to right in top panel). The electrical surface potential on the circular patch is fixed to $\psi_s = -0.9$ (TiO_2 at pH 6), the remaining surfaces are set to $\psi_s = -3$ (SiO_2 at pH 6). A red circle in each image denotes the input feature size. Cross sections of image intensities, along the red dashed line, are shown for all cases (bottom). The study suggests that features as small as 20 nm can be detected by this approach under the stated conditions. (*B*) Calculated signal-to-noise ratio (SNR) across a 500 nm feature for differently charged probe molecules, displaying a maximum value at a gap height of ≈110 nm. SNR clearly increases with increasing probe charge. (*C*) A plot of SNR vs radius of a TiO_2 feature on SiO_2 at a gap height H = 110 nm, corresponding to the maxima in (*B*), shows that the minimum feature size required to achieve SNR >2 corresponds to approximately 2 Debye lengths for c = 0.1 mM NaCl.

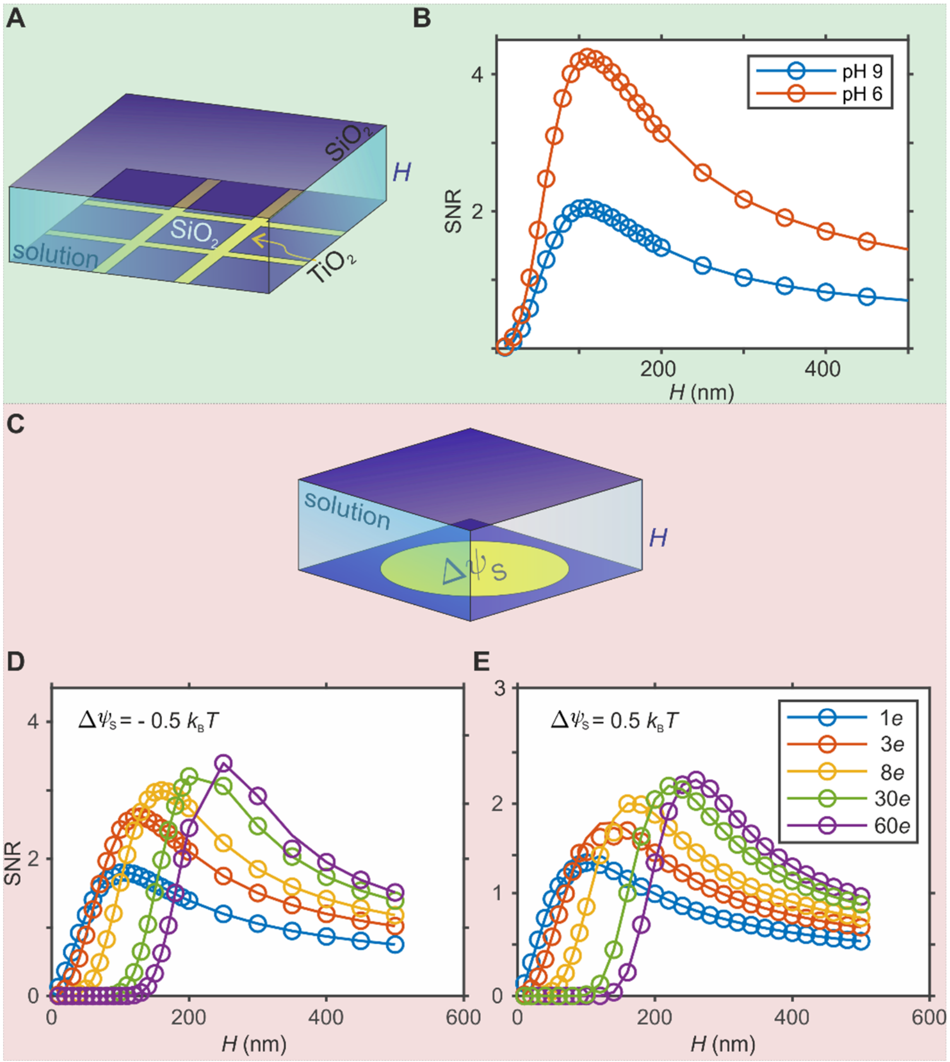

Fig. S11. Simulation-based study of the dependence of measured SNR on solution pH and probe charge for the data shown in Fig. 4*B-D* and Fig. 5*B*. (*A*) Schematic diagram of TiO_2 features on a SiO_2 substrate as measured in Fig. 4 and Fig. 5. (*B*) Calculated SNR in the region of a TiO_2 feature on a SiO_2 substrate (shown schematically in panel (*A*)) at pH6 (orange) and pH9 (blue). Here the SNR is defined as the signal of the feature ($\propto I$, the measured intensity based on Eq. (S8)), divided by the noise on the background signal (from the featureless area). The peak in SNR is expected to occur at a height of $H \approx 110$ nm. (*C*) Schematic of a circular feature of radius 500 nm, surrounded by SiO_2, where $\Delta\psi_s$ is defined as the potential difference between the circular feature region (yellow) and the electrical surface potential of the background SiO_2 region (blue), assuming that the silica has a surface potential $\psi_s = -3.0$ measured at pH 6 and 0.01 mM NaCl. (*D-E*) Expected SNR values as a function of gap height, H, for the feature depicted in (*C*) differently

charged species (q_{eff} = −1, −3, −8, −30, and −60 e) for fixed values of the surface potential difference, $\Delta\psi_{\mathrm{s}}$ = – 0.5 (d) and 0.5 (*E*).

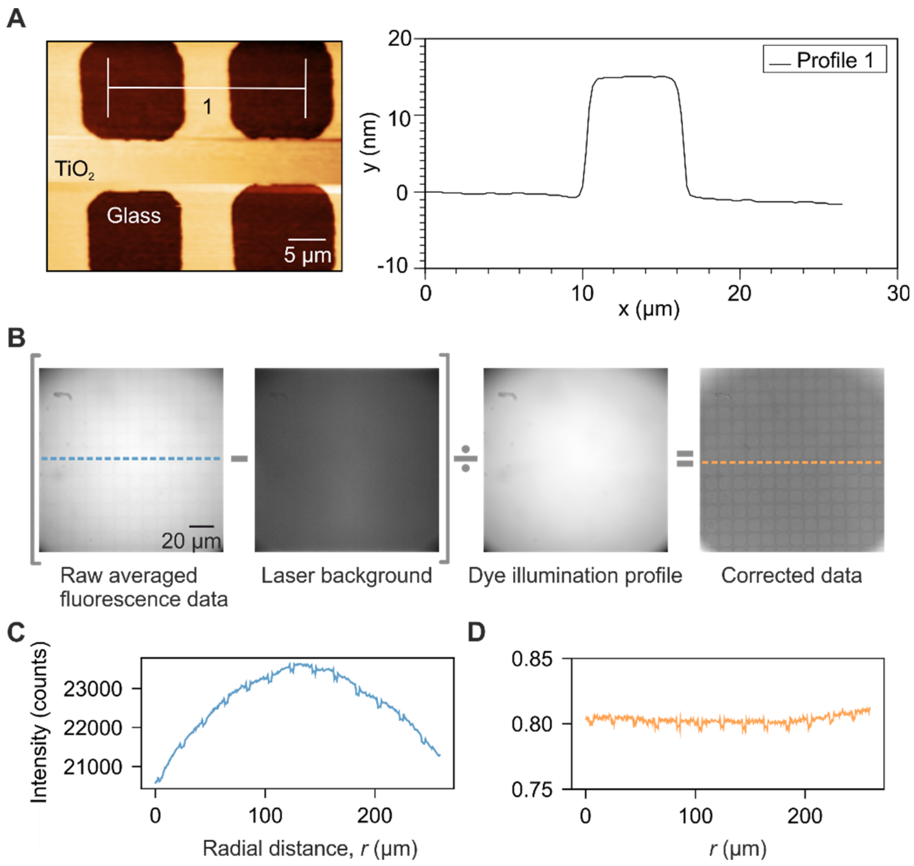

Fig. S12. Image correction and atomic force microscopy (AFM) characterization of TiO_2/SiO_2 patterned substrates. (*A*) AFM image (left) and height profile (right) of TiO_2 thin film on a glass substrate showing a TiO_2 feature height of ≈15 nm. (*B*) Image of a patterned TiO_2/glass substrate showing how the background and illumination profile corrections are performed as previously described in Fig. S1*A*. (*C*) Intensity profile through the raw image shown in (*B*). (*D*) Intensity profile through the corrected image shown in (*C*).

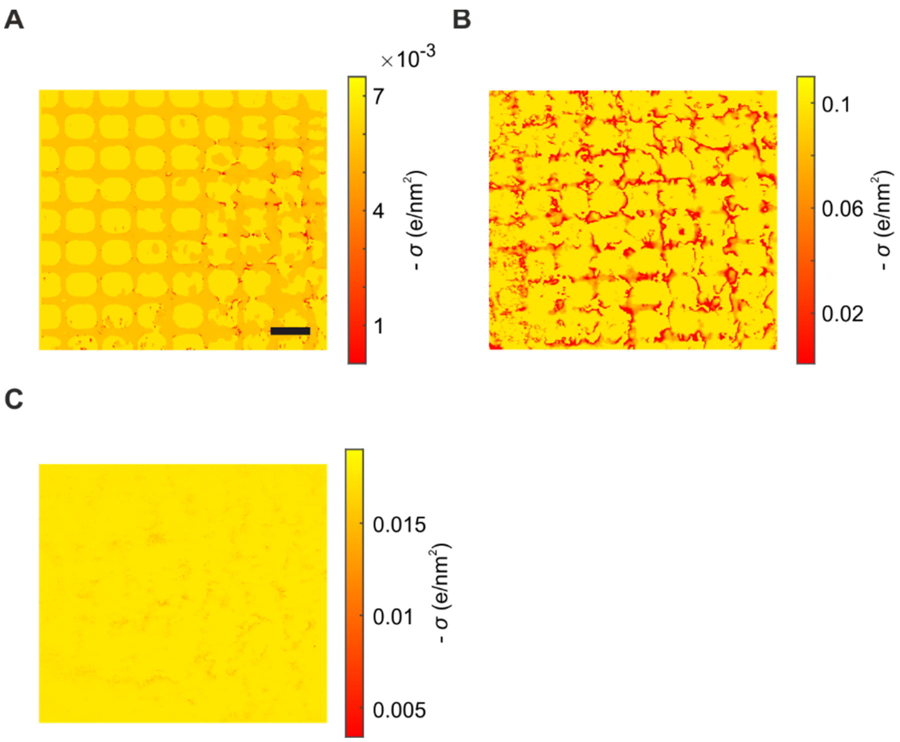

Fig. S13. Electrical surface charge map of TiO_2/SiO_2 patterned substrates. (*A*) Spatial distribution of the electrical surface charge density, σ, on a patterned substrate inferred from the measured optical intensity distribution in the scanning probe system at two different heights $H =$ 100 nm and 500 nm at pH≈6 and low ionic strength (0.1 mM) (Data from Fig. 5). (*B*) Spatial distribution of the electrical surface charge on a patterned substrate at high ionic strength (100 mM) at pH≈6. (*C*) Spatial distribution of the electrical surface charge on a patterned substrate at low ionic strength (0.1 mM) at pH≈9. The scale bar corresponds to 20 μm and is the same for all images. Each image is the charge density counterpart of the corresponding surface electrical potential distribution shown in Fig. 5*E*-*F*.

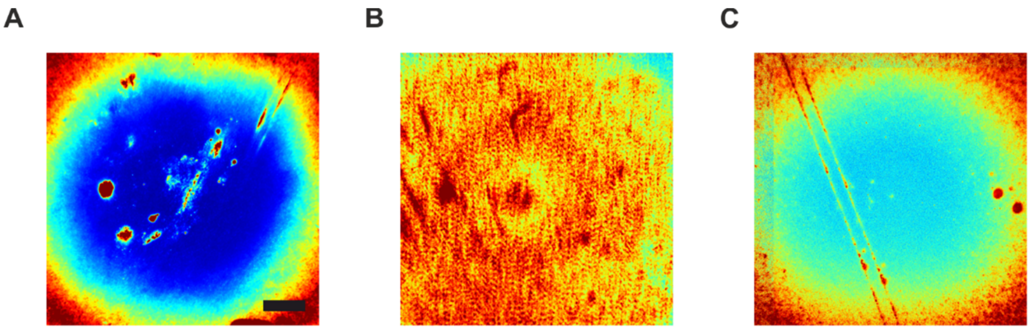

Fig. S14. Examples of images illustrating adsorption of probe molecules on weakly or oppositely charged surfaces. For materials of very low surface charge, or of opposite sign of charge to the probe molecules, or when the solution pH is close to the isoelectric point of the surface, probe molecules may adsorb to the surface generating characteristic optical images which are not suitable for data processing. (*A*) Example of an image obtained in the lens confinement system where sticking of dye molecules to the surface manifest as patchy regions of high intensity in the recorded image. Additionally, the image intensity contours are not radially symmetric due to sticking of dye molecules to the surface. (*B*) Example of an image where the fluorescence intensity distribution does not increase radially from the center, as would be expected in a system where probe molecules are present solely in bulk solution. (*C*) Example of a weakly charged surface where there is substantial sticking of dye molecules to the surface. The resulting intensity is superimposed on a background intensity that increases gradually towards the edges of the field of view, where dye molecules diffusing in the volume contribute to the measured local intensity. Images can also show "punctate" regions of transient high intensity arising from adsorption/desorption of individual dye molecules to the substrate surface. Note that in situations where we do observe evidence of sticking with a weakly charged probe molecule (say $q_{\mathrm{eff}} = -1e$) we do have the option of using a more highly charged probe molecule ($q_{\mathrm{eff}} = -2$ to $-3e$, or higher if needed). This permits us to reliably probe surface electrical properties even in situations where a weakly charged probe molecule shows evidence of adsorption to the surface. The scale bar corresponds to 40 μm and is the same for all images.

Supplementary Movie Legends

Movie S1 (separate file). Time lapse of the moving H+ wave across the field of view of the variable-height gap presented in Fig. 4*D*. The solution changes from pH ≈9.6 to 3.5 as the protons diffuse across the system, altering the electrical charge on the surface and rendering local chemical heterogeneity visible in the form of optical contrast.

Movie S2 (separate file). Height sweep of the flat scanning probe from Fig. 5*E*, using a 0.01 mM NaCl solution at pH6. Due to a difference in the electrostatic surface potential between the materials at pH 6, a strong contrast is observed between the TiO_2 areas (lines surrounding the squares) and the SiO_2 (material inside the squares). At large heights ($H >$ 500 nm), the contrast disappears as the collected fluorescent emission is dominated by dye molecules in the "bulk solution" in the gap that is relatively insensitive to the surface electrical potential.

Movie S3 (separate file). Height sweep of the flat scanning probe from Fig. 5*E*, for an experiment in 100 mM NaCl solution at pH6. Due to differences in the electrostatic surface potential between the materials at pH6, a strong contrast is observed between the TiO_2 areas (lines surrounding the squares) and the SiO_2 (material inside the squares). The contrast is only visible up to small heights $H \approx$ 50 nm, as the length scale of the electrostatic interactions is shorter and given by the Debye length, approximately 1 nm in this case.

Movie S4 (separate file). Height sweep of the flat scanning probe from Fig. 5*F*, using a 0.1 mM NaCl solution at pH9. No contrast is observed between the TiO_2 areas (lines surrounding the squares) and the SiO_2 (material inside the squares), as the electrostatic surface potentials of both materials are similar under these conditions ($|\psi_{\mathrm{s,SiO_2}}| \approx |\psi_{\mathrm{s,TiO_2}}| \approx 3$).

SI References

1. G. T. Morrin, D. F. Kienle, D. K. Schwartz, Standalone interferometry-based calibration of convex lens-induced confinement microscopy with nanoscale accuracy. *Analyst* **144**, 2628-2634 (2019).
2. M. Born, E. Wolf, Principles of Optics, 7th Anniversary Edition. *Principles of Optics, 7th Anniversary Edition*, 1-952 (2019).
3. G. Beadie, M. Brindza, R. A. Flynn, A. Rosenberg, J. S. Shirk, Refractive index measurements of poly(methyl methacrylate) (PMMA) from 0.4-1.6 mu m. *Applied Optics* **54**, F139-F143 (2015).
4. R. von Klitzing, Internal structure of polyelectrolyte multilayer assemblies. *Physical Chemistry Chemical Physics* **8**, 5012-5033 (2006).
5. M. Krishnan, A simple model for electrical charge in globular macromolecules and linear polyelectrolytes in solution. *J Chem Phys* **146**, 205101 (2017).
6. F. Ruggeri *et al.*, Single-molecule electrometry. *Nature Nanotechnology* **12**, 488-495 (2017).
7. R. R. Netz, H. Orland, Variational charge renormalization in charged systems. *European Physical Journal E* **11**, 301-311 (2003).
8. R. Kjellander, J. Ulander, Effective ionic charges, permittivity and screening length: dressed ion theory applied to 1 : 2 electrolyte solutions. *Molecular Physics* **95**, 495-505 (1998).
9. R. Ramirez, R. Kjellander, Dressed molecule theory for liquids and solutions: An exact charge renormalization formalism for molecules with arbitrary charge distributions. *Journal of Chemical Physics* **119**, 11380-11395 (2003).
10. F. Ruggeri, M. Krishnan, Entropic Trapping of a Singly Charged Molecule in Solution. *Nano Letters* **18**, 3773-3779 (2018).
11. M. Oheim, A. Salomon, M. Brunstein, Supercritical Angle Fluorescence Microscopy and Spectroscopy. *Biophysical Journal* **118**, 2339-2348 (2020).
12. E. H. Hellen, D. Axelrod, Fluorescence emission at dielectric and metal-film interfaces. *Journal of the Optical Society of America B-Optical Physics* **4**, 337-350 (1987).
13. W. Lukosz, R. E. Kunz, Light-Emission by Magnetic and Electric Dipoles Close to a Plane Dielectric Interface .2. Radiation-Patterns of Perpendicular Oriented Dipoles. *Journal of the Optical Society of America* **67**, 1615-1619 (1977).
14. J. Enderlein, T. Ruckstuhl, S. Seeger, Highly efficient optical detection of surface-generated fluorescence. *Applied Optics* **38**, 724-732 (1999).
15. B. W. Ninham, V. A. Parsegian, Electrostatic Potential between Surfaces Bearing Ionizable Groups in Ionic Equilibrium with Physiologic Saline Solution. *Journal of Theoretical Biology* **31**, 405-428 (1971).
16. K. Bohinc, A. Shrestha, M. Brumen, S. May, Poisson-Helmholtz-Boltzmann model of the electric double layer: Analysis of monovalent ionic mixtures. *Physical Review E* **85**, 031130 (2012).
17. D. Ben-Yaakov, D. Andelman, R. Podgornik, D. Harries, Ion-specific hydration effects: Extending the Poisson-Boltzmann theory. *Current Opinion in Colloid & Interface Science* **16**, 542-550 (2011).
18. H. F. Fan, F. P. Li, R. N. Zare, K. C. Lin, Characterization of two types of silanol groups on fused-silica surfaces using evanescent-wave cavity ring-down spectroscopy. *Analytical Chemistry* **79**, 3654-3661 (2007).
19. A. M. Darlington, J. M. Gibbs-Davis, Bimodal or Trimodal? The Influence of Starting pH on Site Identity and Distribution at the Low Salt Aqueous/Silica Interface. *The Journal of Physical Chemistry C* **119**, 16560-16567 (2015).
20. C. Macias-Romero, I. Nahalka, H. I. Okur, S. Roke, Optical imaging of surface chemistry and dynamics in confinement. *Science* **357**, 784-788 (2017).
21. S. W. Ong, X. L. Zhao, K. B. Eisenthal, Polarization of water-molecules at a charged interface - 2nd harmonic studies of the silica water interface. *Chemical Physics Letters* **191**, 327-335 (1992).

22. S. H. Behrens, D. G. Grier, The charge of glass and silica surfaces. *Journal of Chemical Physics* **115**, 6716-6721 (2001).
23. ATTO-TEC (2019) Fluorescent Labels and Dyes. (Germany).
24. L. Belloni, Ionic condensation and charge renormalization in colloidal suspensions. *Colloids and Surfaces a-Physicochemical and Engineering Aspects* **140**, 227-243 (1998).
25. G. S. Manning, Electrostatic free energies of spheres, cylinders, and planes in counterion condensation theory with some applications. *Macromolecules* **40**, 8071-8081 (2007).
26. M. Aubouy, E. Trizac, L. Bocquet, Effective charge versus bare charge: an analytical estimate for colloids in the infinite dilution limit. *Journal of Physics a-Mathematical and General* **36**, 5835-5840 (2003).
27. G. Gonella *et al.*, Water at charged interfaces. *Nature Reviews Chemistry* **5**, 466-485 (2021).
28. M. A. Brown *et al.*, Determination of Surface Potential and Electrical Double-Layer Structure at the Aqueous Electrolyte-Nanoparticle Interface. *Physical Review X* **6**, 011007 (2016).
29. R. W. Obrien, L. R. White, Electrophoretic Mobility of a spherical colloidal particle. *Journal of the Chemical Society-Faraday Transactions II* **74**, 1607-1626 (1978).
